# Rapid Characterizaiton of Chemical Constituents of the Tubers of *Gymnadenia conopsea* by UPLC–Orbitrap–MS/MS Analysis

**DOI:** 10.3390/molecules25040898

**Published:** 2020-02-18

**Authors:** Xin Wang, Xiang-Jian Zhong, Na Zhou, Ning Cai, Jia-Hui Xu, Qing-Bo Wang, Jin-Jie Li, Qian Liu, Peng-Cheng Lin, Xiao-Ya Shang

**Affiliations:** 1 Beijing Key Laboratory of Bioactive Substances and Functional Foods, Beijing Union University, Beijing 100191, China; shtwangxin@buu.edu.cn (X.W.); xiangjzhong@163.com (X.-J.Z.); zhouna163163@163.com (N.Z.); caining1225@163.com (N.C.); jsclefaxu@yeah.net (J.-H.X.); wangqingbo17@163.com (Q.-B.W.); lijinjie.7785004@163.com (J.-J.L.); shtliuqian@buu.edu.cn (Q.L.); 2 Qinghai Provincial Key Laboratory of Phytochemistry for Tibetan Plateau, Qinghai University for Nationalities, Xining 810000, China; qhlpc@126.com

**Keywords:** *Gymnadenia conopsea*, UPLC–Orbitrap–MS/MS, chemical constituents, rapid characterization

## Abstract

*Gymnadenia conopsea* R. Br. is a traditional Tibetan medicinal plant that grows at altitudes above 3000 m, which is used to treat neurasthenia, asthma, coughs, and chronic hepatitis. However, a comprehensive configuration of the chemical profile of this plant has not been reported because of the complexity of its chemical constituents. In this study, a rapid and precise method based on ultra-high performance liquid chromatography (UPLC) combined with an Orbitrap mass spectrometer (UPLC–Orbitrap–MS/MS) was established in both positive- and negative-ion modes to rapidly identify various chemical components in the tubers of *G. conopsea* for the first time. Finally, a total of 91 compounds, including 17 succinic acid ester glycosides, 9 stilbenes, 6 phenanthrenes, 19 alkaloids, 11 terpenoids and steroids, 20 phenolic acid derivatives, and 9 others, were identified in the tubers of *G. conopsea* based on the accurate mass within 3 ppm error. Furthermore, many alkaloids, phenolic acid derivates, and terpenes were reported from *G. conopsea* for the first time. This rapid method provides an important scientific basis for further study on the cultivation, clinical application, and functional food of *G. conopsea.*

## 1. Introduction

*Gymnadenia conopsea* R. Br. is a perennial herb belonging to the Orcidaceae family and is widely distributed in Tibet, Xinjiang, Qinghai, Gansu, and Sichuan in China [1]. The tubers of this plant are similar to the palm of the human hand, so was given the Chinese name “shou zhang shen”. *G. conopsea* has widely been used as a traditional Tibetan remedy and traditional health food for the treatment of neurasthenia, asthma, coughs, and chronic hepatitis [2,3,4]. In recent years, modern pharmacological experiments have demonstrated that the ethanol extract or fractions obtained from the tubers of *G. conopsea* have effects on Alzheimer’s disease and are anti-viral [5,6,7]. A number of previous studies have reported the isolation and structural determination of different categories in this plant, including glucosyloxybenzyl-2-isobutylmalates, phenanthrenes, and stilbenes [8]. however, traditional separation and identification methods require a large amount of materials and take a long time, and only the main components can be obtained, which do not fully explain the chemical profile of this plant. At the same time, the resources of this plant are rare and blind separation is a waste of resources. A comprehensive configuration of the chemical profile of *G. conopsea* could be used as guidance for further study of active components, and also could save resources. Therefore, a rapid and sensitive method to figure out the chemical components in the tubers of *G. conopsea* was urgently needed.

A rapid, efficient, and precise method focused on identification of chemical components is very important for complex herb medicines. Recently, based on the highly efficient separation performance of ultra-high performance liquid chromatography (UPLC) and high sensitivity of mass spectrometry (MS), UPLC coupled with high-resolution mass spectrometry (HRMS) has become an important tool for characterization of chemical components in natural products [9]. Furthermore, a combination of UPLC separation with an Orbitrap MS system (UPLC–Orbitrap–MS/MS) has been widely used for screening and identification of chemical components in herbal medicines because of the advantages in terms of the peak capacity, resolution, separation time, and detection sensitivity [10,11,12].

In this study, a method based on UPLC–Orbitrap–MS/MS was established for rapid and sensitive characterization of various chemical components in the tubers of *G. conopsea* for the first time. A total of 91 components belonging to seven categories in the tubers of *G. conopsea* were identified in a short time, which will provide a basis for further study of the relationship between the constituents and pharmacology.

## 2. Results and Discussion

### 2.1. Optimization of Ultra-High Performance Liquid Chromatography (UPLC) and Mass Spectrometry (MS) Conditions

In order to obtain the optimal elution conditions for the separation and analytical sensitivity of constituents, a series of parameters (mobile phase, flow rate, and column temperature) were investigated. According to the previous reports [13], there are many glycoside compounds in the tubers of *G. conopsea*. A comparative study based on the chromatographic separation and detection sensitivity revealed that the best performance was achieved with methanol as the organic part of the mobile phase. Due to the compounds containing carboxyl and phenolic hydroxyl, the moiety was tailed on the C18 column, which could be improved by adding a small amount of organic acid. The alkaloid compounds generally showed better mass spectrometric responses in positive ionization mode. Therefore, it was finally decided that methanol/0.1% formic acid aqueous solution was used as the mobile phase. Finally, a column temperature of 40 °C and a flow rate of 0.3 mL/min were set to reduce the pressure and obtain better separation.

Some parameters of heated electrospray ionization (HESI) sources (spray voltage, source heater temperature, capillary temperature, sheath gas flow, auxiliary gas flow, capillary voltage, and S-lens voltage) were also optimized to obtain high sensitivity for most compounds. The optimal conditions were set as follows: spray voltage, 4 kV/3.5 kV (positive/negative); source heater temperature, 350 °C; capillary temperature, 350 °C; sheath gas flow, 50 arb; auxiliary gas flow, 10 arb; and S-lens RF level, 50. The mass scan range was set at *m*/*z* 150–2250 Da in the full scan mode, and the resolution was set at 70,000. To acquire the more abundant MS/MS2 spectrum, the MS/MS energy was set at 20, 40, and 60 V as stepped normalized collision energy (NCE) and the resolution was set at 17,500.

### 2.2. Identification of Main Constituents in G. conopsea Extract

**Figure 1:**
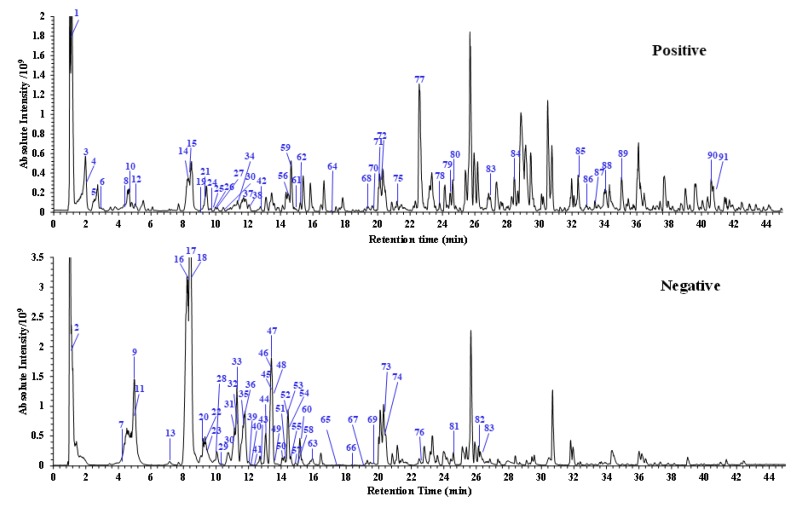
The total ion chromatograms of the tubers of *G. conopsea*, extracted by ultra-high performance liquid chromatography combined with an Orbitrap mass spectrometer (UPLC–Orbitrap–MS/MS) in positive- and negative-ion modes.

The total ion chromatogram (TIC) of *G. conopsea* extract in positive- and negative-ion modes are shown in Figure 1. A total of 91 chemical constituents were identified, including 17 succinic acid ester glycosides, 9 stilbenes, 6 phenanthrenes, 19 alkaloids, 11 terpenoids and steroids, 20 phenolic acid derivatives, and 9 others (the chemical structures and MS2 spectra of some constituents see Figure S1–S41). The compounds identification process contained many steps. Firstly, the analysis data were imported into the Compound Discoverer 2.1 software (The workflow tree see Figure S42), which includes the OTCML database and the free chemical structure database, including Massbank, NIST, ChemSpider, and mzCloud. The chemical elemental composition for each target peak was accurately assigned within a mass error of 3 ppm. Then, the formulas that were obtained from Compound Discovery were searched in the self-built chemical database of *gymnadenia* to match the known structures in this genus. For those formulas not included in this genus, we referred to the database search results for confirmation. Then, the fragment ions were used to further confirm the chemical structures. The retention time, compound name, formula, *m*/*z* values of adduct ions and MS/MS fragment ions in positive/negative ESI modes, mass error, and accurate molecular mass are shown in Table 1.

#### 2.2.1. Succinic Acid Ester Glycosides

Succinic acid ester glycosides were the main components in *G. conopsea*, which consisted of succinic acid, glycosyl, and a benzyl moiety. A total of 17 succinic acid ester glycosides were identified in the tubers of *G. conopsea* extract, and the deprotonated molecules [M − H]^−^ were found in the ESI–MS spectra for all compounds. All the esters glycosides could be classified into glycosyloxybenzyl 2-isobutylmalate and glycosyloxybenzyl 2-isobutyltartrate. In tandem mass spectra of succinic acid ester glycosides, the losses of H_2_O, COOH and C_6_H_10_O_5_ (glycose moiety), and C_13_H_17_O_7_ (glycosyloxybenzyl moiety) are commonly observed.

**Figure 2:**
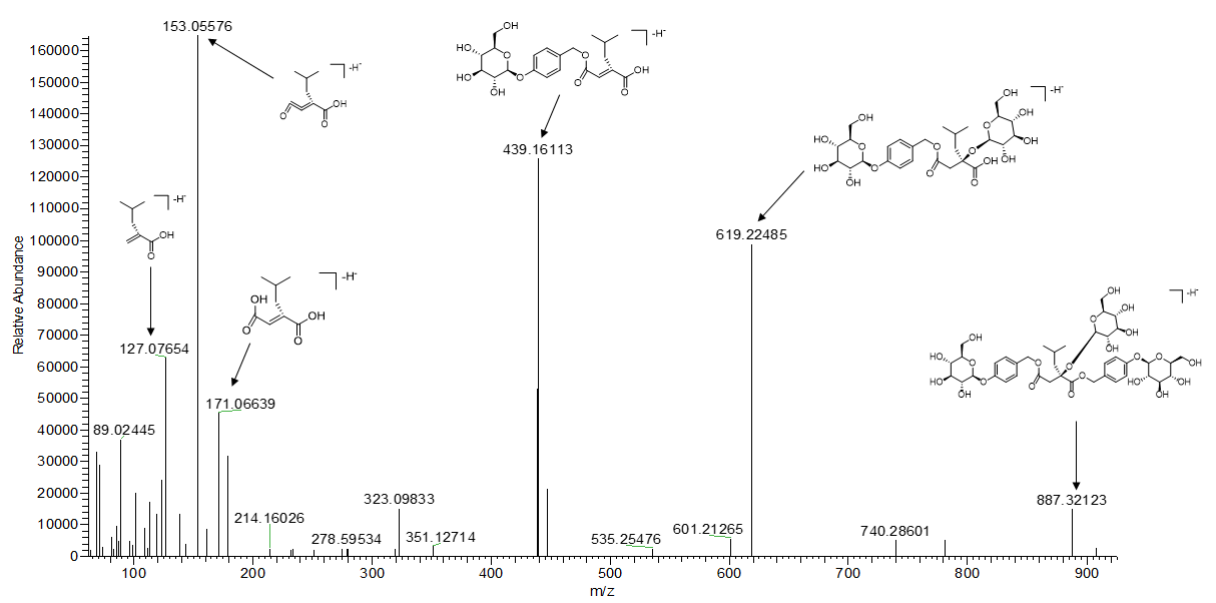
The possible fragmentation mechanism of dactylorhin A.

Compounds **16**, **29**, **31**, **36**, **44**, **47**, **48**, and **52**–**55** were glycosyloxybenzyl 2-isobutylmalate. Among them, compound **16** showed a [M-H]^−^ ion at *m*/*z* 351.12982, and gave fragment ions at 351.12982 179.05595, 171.06635, and 127.07648 corresponding to [M-H]^−^, [M-H-C_6_H_10_O_5_]^−^, [M-H-C_6_H_10_O_5_-H_2_O]^−^, and [M-H-C_6_H_10_O_5_-H_2_O-COOH]^−^, respectively; this compound was tentatively identified as dactylorhic C [15]. Except for **16**, all other compounds had the glycosyloxybenzyl moiety and had similar fragmentation patterns. Taking compound **47** as an example, it had a [M-H]^−^ ion at *m*/*z* 887.32123. The fragment ion *m*/*z* 619.22485 [M-H-C_13_H_16_O_6_]^−^ was easily produced, which indicated that the glucopyranosyloxy-benzyl moiety was easily lost. Then, the fragment ion *m*/*z* 439.16113 [M − H − C_13_H_16_O_6_ − C_6_H_10_O_5_]^−^, with its high relative abundance, was easily produced from *m*/*z* 619.22485 by neural loss of the glycose moiety at C_2_–OH. Fragment ions *m*/*z* 323.09833, 171.06639, 153.05572, and 127.07654 were derived from the malate moiety by the loss of H_2_O and COOH. Compared with the literature data, compound **47** was identified as dactylorhin A [15]. The possible fragmentation mechanism of dactylorhin A is depicted in Figure 2. In a similar way, the other nine compounds were identified according to their molecular mass, formula, MS/MS fragments, and related literature studies, including grammatophylloside C (**29**) [16], coelovirin B (**31**) [14], dactylorhin E (**36**) [15], coelovirins A (**44**) [14], gymnoside II (**48**) [15], gymnoside III (**52**) [5], gymnosides VII (**53**) [5], gymnoside I (**54**) [14], and militarine (**55**) [17].

**Figure 3:**
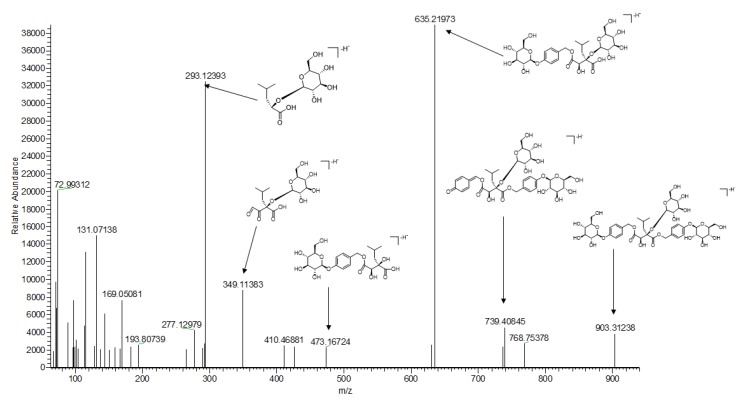
The possible fragmentation mechanism of dactylorhin B.

Compounds **9**, **28**, **32**, **33**, **35**, and **46** were glycosyloxybenzyl 2-isobutyltartrates. The [M − H]^−^ ion of compound **9** was shown at *m*/*z* 367.12473. Its MS2 fragment ions at *m*/*z* 293.12454 [M − H − C_2_H_2_O_3_]^−^, 187.06120 [M − H − C_6_H_12_O_6_]^−^, 143.07137 [M − H − C_6_H_12_O_6_ − CO_2_]^−^, and 99.08157 [M − H − C_6_H_12_O_6_ − CO_2_ − CO_2_]^−^ were characteristic fragments of the tartrate moiety. All except compounds **9** have the same fragment of the glucopyranosyloxy-benzyl moiety (285 Da). Compounds **28**, **32**, **33**, **35**, and **46** showed a [M-H]^−^ ion at *m*/*z* 635.21948, 1065.37610, 903.31238, 741.26056, and 487.18188. They have similar fragmentation patterns, including ions at *m*/*z* 349.11383, 293.12393, and 277.12915, which were identified as coelovirins D [14], (−)-(*2R*,*3S*)-1-(4-β-d-glucopyranosyloxybenzyl)-2-*O*-β-d-glucopyranosyl-4-{4-[α-d-glucopyranosyl-(1-4)-β-d-glucopyranosyloxy]benzyl}-2-isobutyltartrate [4], dactylorhin B [4], loroglossin [17], and (−)-(*2R*,*3S*)-1-(4-β-d-glucopyranosyloxybenzyl)-4-methyl-2-isobutyltartrate [4], respectively. The possible fragmentation mechanism of dactylorhin B (**33**) is depicted in Figure 3.

#### 2.2.2. Stilbenes

Stilbenes were structures containing one or more C6-C2-C6 units, which were widely distributed in medicinal plants. A total of eight stilbenes in the tubers of *G. conopsea* extract were identified in positive and negative ion modes. According to their molecular mass, formula, MS/MS fragments, and related literature studies, compounds **38**, **39**, **40**, **57**, **64**, **69**, **72**, **73**, and **76** were considered to be isorhapontigenin [18], rhaponticin [19], piceatannol [20], dihydro-resveratrol [21], batatasin III [22], 3,3′-dihydroxy-4-(4-hydroxybenzyl)-5-methoxybibenzyl [23], bulbocodin C [24], bulbocodin D [24], and 3,3′-dihydroxy-2,6-bis(4-hydroxybenzyl)-5-methoxybibenzyl [25], respectively.

**Figure 4:**
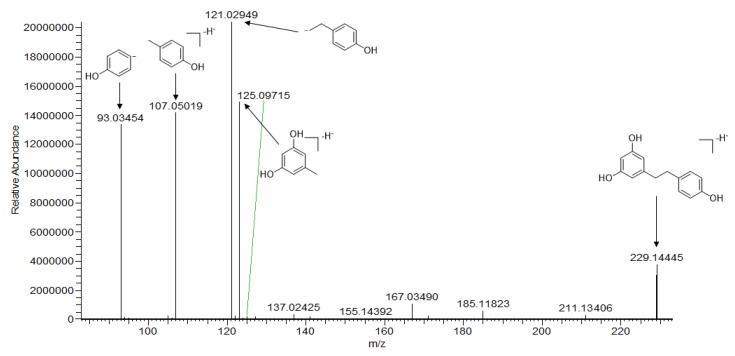
The possible fragmentation mechanism of dihydro-resveratrol.

Taking compound **57** as an example, it had a [M − H]^−^ ion at *m*/*z* 229.14445, and the highest relative abundance ion *m*/*z* 121. 02949 [M − H − C_6_H_4_O_2_]^−^ was easily yielded by the breakage of the C2-chain. The fragments ions at *m*/*z* 123.04515, 107.05019, and 93.03454 were formed in the same fragmentation pattern. Its fragmentation process was the same as in the literature and was identified as dihydro-resveratroll [21]. The possible fragmentation mechanism of compound **57** is depicted in Figure 4.

#### 2.2.3. Phenanthrenes

Six phenanthrenes were identified from the extract of the *G. conopsea* extract, including 1-((4-hydroxyphenyl)methyl)-4-methoxy-2,7-phenanthrenediol (**71**) [26], gymconopin A (**74**) [26], 9,10-dihydro-2-methoxy-4,5-phenanthrenediol (**75**) [26], blestriarene A (**82**) [26], gymconopin (**83**) [26], and blestriarene B (**84**) [26].

**Figure 5:**
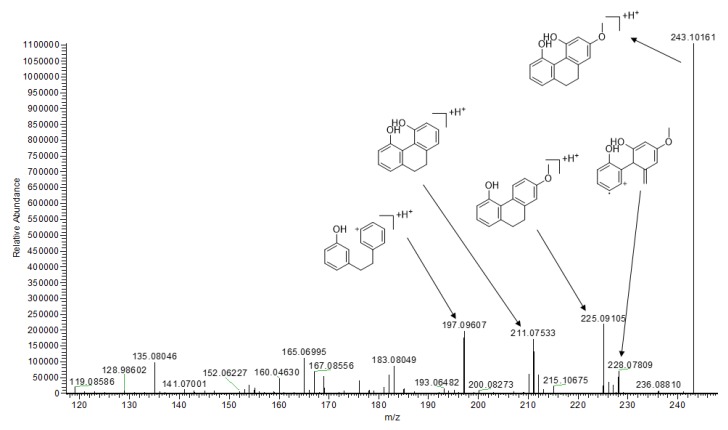
The possible fragmentation mechanism of 9,10-dihydro-2-methoxy-4,5-phenanthrenediol.

A typical phenanthrene, 9,10-dihydro-2-methoxy-4,5-phenanthrenediol (**75**), was taken as an example to investigate the MS/MS fragmentation pattern of this type of compound in *G. conopsea*. The protonated molecular ion of compound **75** was *m*/*z* 243.10161 [M + H]^+^ in positive ESI mode, and its dehydration of C11–OH yielded the fragment ion *m*/*z* 225.09105 [M + H − H_2_O]. The fragment ion *m*/*z* 211.07533 [M + H − OCH_3_]^+^ was produced by the loss of methoxy at C-13. Then, the continuous dehydration and breakage of the C-ring formed the fragment ion *m*/*z* 197.09607 (Figure 5).

#### 2.2.4. Phenolic Acid derivatives

Phenolic acids were structures containing one or more phenolic hydroxyl moieties, which were widely distributed in medicinal plants. A total of 20 phenolic acid derivates in the tubers of *G. conopsea* extract were identified in negative and positive ion modes. Among them, compounds **7**, **11**, **13**, **17**, and **18** were aromatic glycosides. The loss of hexose residues (glycose 162 Da, rhamnose 146Da) was often seen in these compounds. Taking compound **7** as an example, the deprotonated molecular ion *m*/*z* 447.15176 was detected in the spectrum. Fragment ion *m*/*z* 341.10901 [M − H − 106]^−^ with the highest relative abundance was easily produced from *m*/*z* 447.15176 [M − H]^−^ by cleavage of the glycoside band. The fragment ions *m*/*z* 179.05614 and 161.04562 were glycose moieties. Compounds **21**–**23** and **25** were phenylpropanoids, which were considered to be isoferulic acid, ferulic acid, *p*-coumaric acid, and (E)-4-Methoxycinnamic acid [30,31]. There were four flavonoid glycosides and five flavonoids, which were identified as quercetin-3β-d-glucoside (**45**) [34], cirsimarin (**49**) [35], astragalin (**50**) [36], kaempferol-7-*O*-glucoside (**56**) [37], desmethylxanthohumol (**59**) [38], isorhamnetin (**61**) [39], naringenin chalcone (**63**) [40], equol (**65**) [41], and galangin (**82**) [42], respectively.

#### 2.2.5. Alkaloid

A total of 19 alkaloids were identified from the extract of *G. conopsea*, including amino acids, adenosine, indoles, cyclic peptides, and amides. As depicted in Table 1, in positive ion mode, compounds **3**, **6**, **12**, and **26** were considered as adenosine [43], *N*-(4-methyoxyphenyl)-1H-pyrazolo[3,4-d]pyrimidin [46], 5′-*S*-Methyl-5′-thioadenosine [49], and *N*-(4-hydroxybenzy)-adenine-riboside [53], respectively. Taking compound **6** as an example, it had a [M + H]^+^ ion at *m*/*z* 242.10341 in the spectrum. Two main fragment ions at *m*/*z* 136.06171 and 107.04944 were obviously observed. Among them, the most abundant fragment ion *m*/*z* 136.06171 was suggested by the loss of the phenol residue [M + H − 107]^+^. The fragment ion at *m*/*z* 107.04944 was identified as purine. Compared to the MS spectra data and references, compound **6** was tentatively identified as *N*-(4-methyoxyphenyl)-1H-pyrazolo[3,4-d] pyrimidin [46].

Compounds **19** and **24** had similar fragmentation behavior and showed [M + H]^+^ ions at *m*/*z* 327.13342 and 211.14403, respectively. According to reference mass spectra and fragmentation spectra reported in the literature studies, two cyclic peptides were identified as cyclo (tyrosy-tyrosyl) [6] and cyclo (leucylprolyl) [52] in the tubers of *G. conopsea.* The other 13 alkaloids were identified according to their molecular mass, formula, MS/MS fragments, and related literature studies, which are shown in Table 1.

#### 2.2.6. Terpenoid and Steroid

Terpenoids and steroids were derived from methylglutaric acid (MWA). Eleven terpenoids and steroids were identified in this study, including one sesquiterpenoid, one diterpenoid, four triterpenoids, and five steroids. Compound **51** had [M − H]^−^ ion at *m*/*z* 263. 12869, and its fragments were at *m*/*z* 219.13905 [M-H-COO]^−^, 204.11546 [M-H-COO-CH_2_]^−^, 201.12842 [M − H − COO − H_2_O]^−^, and 151.07640 [M − H − C_6_H_8_O_2_]^−^. Its fragmentation process was the same as the literature and identified as abscisic acid [60].

In tandem mass spectra of terpenoids and steroids in this plant, the neutral losses of H_2_O (18 Da) and CO (28 Da) are commonly observed. Compounds **77**, **87**, **89**, and **90** were triterpenoids, which gave [M + H]^+^ ions at *m*/*z* 489.35718, 425.37735, 411.36194, and 427.39322, respectively. Thus, they were (3β,5α,9α)-3,6,19-trihydroxyurs-12-en-28-oic acid [60], lupenone [65], 4,4-dimethyl-5α-cholesta-8,14,24-trien-3β-ol [67], and lupeol [68]. Compound **88** was taken as an example to investigate the MS/MS fragmentation pattern of this type of compound in *G. conopsea*. The protonated molecular ion of compound **88** was *m*/*z* 413.37762 [M + H]^+^ in positive ESI mode, and its dehydration of C3-OH with the adjacent hydrogen easily yielded the fragment ion *m*/*z* 395.36703 [M + H − 18]^+^. The following fragmentation pattern of fragment *m*/*z* 395.36703 was the breakage of the side chain to produce the fragment *m*/*z* 255.21051 [M + H − 158]^+^. This was consistent with the literature, and the fragment was identified as poriferasterol [66].

#### 2.2.7. Others

Aside from those listed above, another 9 compounds, namely compounds **2**, **20**, **60**, **66**–**68**, and **80**, were considered to be citric acid [70], succinic acid [71], pinoresinol [72], benzyl-[(6-oxo-7,8,9,10-tetrahydro-6H-benzo[c]chromen-3-yl)oxy]-acetate [72], aloeresin A [73], frangulin B [74], cleomiscosin A [75], bis(methylbenzylidene)sorbitol [75], and umbelliferone [33], respectively. As a typical representative, the MS/MS fragmentation of citric acid was firstly investigated. Its deprotonated molecular ion was *m*/*z* 191.01979 [M − H]^−^ in negative ESI mode, and its main fragmentation pattern was 173.00919 [M − H − 18]^−^. The fragment *m*/*z* 129.01920 [M − H − 62]^−^ was yielded through decarboxylation and dehydration. The most abundant fragment ion *m*/*z* 111.00877 [M − H − 80]^−^ was produced from the fragment *m*/*z* 129.01920.

## 3. Materials and Methods

### 3.1. Chemicals and Reagents

Methanol, acetonitrile, and formic acid (all MS grade) were purchased from Fisher Scientific (Fisher Scientific, Pittsburgh, PA, USA). Dimethyl sulfoxide (DMSO, HPLC grade) was purchased from Sigma-Aldrich (Sigma-Aldrich, St. Louis, MO, USA). The ultra-pure water was purified by a Milli-Q ultrapure water system (Merck Millipore, Milford, MA, USA). All other regents used were of at least analytical grade.

### 3.2. Materials and Sample Preparation

The tubers of *G. conopsea* were collected in Xining City, Qinghai province, China, in August 2018. A botanical voucher specimen of this plant was preserved at the authors’ laboratory and was identified by Professor Pengcheng Lin of Qinghai University for Nationalities.

First, 1.0 g aliquots of the tuber powders were weighed and transferred into a 100 mL Erlenmeyer flask. Next, 50 mL of 95% aqueous methanol solution was added, and then extracted ultrasonically for 1 h. Then, the fluid was filtered and concentrated under reduced pressure in a rotary evaporator. Subsequently, the concentrated extract was dissolved in methanol. Then, the above herb extract solution was filtered through a 0.22 μm PTFE membrane as the sample.

### 3.3. UPLC–Orbitrap–MS/MS

The UPLC separation was carried out on a Thermo Vanquish Flex Binary RSLC platform (Thermo Fisher Scientific, Waltham, MA, USA) equipped with a diode array detector (DAD). Chromatographic separation was conducted on a Thermo Accucore aQ C18 (150 × 2.1 mm, 2.6 μm; Thermo Fisher Scientific, Waltham, MA, USA) kept at 40 °C. The 0.1% formic acid aqueous solution (*v*/*v*, A) and methanol (B) were used as the mobile phase. The gradient elution with a flow rate of 0.3 mL/min was performed as follows: 6–20% B at 0–5 min, 20–21% B at 5–6 min, 21–30% B at 6–7 min, 30–34% B at 7–10 min, 34–40% B at 10–11 min, 40–57% B at 11–17 min, 57–65% B at 17–18 min, 65–90% B at 18–30 min, 90–97% B at 30–37 min, 97–100% B at 37–45 min. The injection volume was set at 2 μL.

The UPLC–Orbitrap–MS/MS detection was conducted on a Q Exactive Plus mass spectrometer (Thermo Fisher Scientific, Waltham, MA, USA). The MS analysis was carried out by the ESI source in both positive- and negative-ion modes and the specific parameters were set as mentioned above. In the MS/MS experiments, the five most intensive ions from each full MS scan were selected for MS/MS fragmentation. The UPLC–MS/MS data were analyzed using Xcalibur 4.1 software (Thermo Fisher Scientific, Waltham, MA, USA), Compound Discoverer 2.1 (Thermo Fisher Scientific, Waltham, MA, USA) loaded with OTCML database 1.0 (Thermo Fisher Scientific, Waltham, MA, USA) and Mass Frontier (Thermo Fisher Scientific, Waltham, MA, USA) were employed to process the UPLC–MS data.

## 4. Conclusions

In this study, an UPLC–Orbitrap–MS/MS approach was firstly developed and applied for rapid separation and identification of the main chemical constituents in the tubers of *G. conopsea*. Based on the high separation speed of UPLC, accurate MS data, and the fragment ion identification strategy, a total of 91 compounds, including 17 succinic acid ester glycosides, 9 stilbenes, 6 phenanthrenes, 19 alkaloids, 11 terpenoids and steroids, 20 phenolic acid derivatives, and 9 others, were identified by comparison of their accurate masses, fragment ions, retention times, and literature studies. Many compounds, such as alkaloids and terpenoids, were reported for *G. conopsea* for the first time. According to the types of compounds identified from this plant, several low polar compounds were identified, which are worthy of further study. This rapid method provides an important scientific basis for further study on the cultivation, clinical application, and functional food of *G. conopsea.*

## Figures and Tables

**Table 1:** All the identified components from *G. conopsea* extract and their ultra-high performance liquid chromatography mass spectrometer (UPLC–MS/MS) data.

No	R.T. (min)	Compound Name	Formula	Exact Mass	Error (ppm)	Adduct Ion (*m*/*z*)	MS2 Fragment (*m*/*z*)	Ref.
**Succinic Acid Ester Glycosides**								
**9**	4.605	coelovirins E	C_14_H_24_O_11_	368.13181	−0.14	367.12473 [M − H]^−^	293.12454, 187.06120, 143.07137 ^a^, 99.08157	[14]
**16**	8.430	dactylorhin C	C_14_H_23_O_10_	352.13690	0.09	351.12982 [M − H]^−^	179.05595, 171.06635,127.07648 ^a^	[15]
**28**	10.072	coelovirins D	C_27_H_40_O_17_	636.22664	0.15	635.21948 [M − H]^−^	349.11404 ^a^, 293.12415, 277.12915,143.07129	[14]
**29**	10.308	grammatophylloside C	C_24_H_28_O_12_	508.28186	2.09	507.14993 [M − H]^−^	221.04546,203.03497 ^a^, 177.05568, 149.06070, 107.05019	[16]
**31**	10.748	Coelovirin B	C_21_H_30_O_12_	474.17371	0.63	473.16614 [M − H]^−^	367.12451, 293.10284, 187.06094, 159.06616,143.0729, 115.07640, 99.08151 ^a^	[14]
**32**	11.08	(−)-(*2R*,*3S*)-1-(4-β-d-glucopyranosyloxybenzyl)-2-*O*-β-d-glucopyranosyl-4-{4-[α-d-glucopyranosyl-(1-4)-β-d-glucopyranosyloxy]-benzyl}-2-isobutyltartrate	C_46_H_66_O_28_	1066.37406	−0.06	1065.37610 [M − H]^−^	797.27228 ^a^, 635.21936, 455.17773, 293.12411	[4]
**33**	11.291	dactylorhin B	C_40_H_56_O_23_	904.32147	1.42	903.31238 [M + H] ^+^	739.40845, 635.21973 ^a^, 473.16724, 349.11383, 293.12393	[15]
**35**	11.678	loroglossin	C_34_H_46_O_18_	742.26858	0.04	741.26056 [M − H]^−^	455.15555, 285.09799, 349.11484, 277.12958 ^a^, 187.09761, 123.04520	[17]
**36**	11.756	dactylorhin E	C_27_ H_40_ O_16_	620.23185	−0.34	619.22369 [M − H]^−^	439.16074, 285.09821, 179.05609,153.05569 ^a^	[15]
**44**	13.063	coelovirins A	C_21_H_30_O_11_	458.17903	0.49	457.17169 [M − H]^−^	285.09793, 189.07683, 171.06650,153.05566, 127.07648 ^a^ 123.04527	[14]
**46**	13.420	(−)-(*2R*,*3S*)-1-(4-β-d-glucopyranosyloxybenzyl)-4-methyl 2-isobutyltartrate	C_22_H_32_O_12_	488.18950	0.25	487.18188 [M − H]^−^	189.07649, 171.06628, 153.05579, 129.09218 ^a^, 99.08157	[4]
**47**	13.420	dactylorhin A	C_40_H_56_O_22_	888.32675	1.49	887.32123 [M − H]^−^	619.22485,439.16113, 323.09833, 153.05572 ^a,^ 171.06639, 127.07654	[15]
**48**	13.425	gymnoside II	C_21_H_30_O_11_	458.17897	0.35	457.17175 [M − H]^−^	285.09827,171.06633, 153.05576, 127.07654,123.04524, 99.08158	[15]
**52**	14.412	gymnoside III	C_42_H_58_O_23_	930.33937	−1.11	929.33154 [M − H]^−^	661.23553, 619.22565 481.17163, 439.16144, 153.05579 ^a^	[5]
**53**	14.431	gymnosides VII	C_50_H_62_O_24_	1046.36365	1.21	1045.35632 [M − H]^−^	741.26141, 635.21967, 455.15485, 349.11420, 293.12424 ^a^	[5]
**54**	14.436	gymnoside I	C_21_H_30_O_11_	458.17897	0.35	457.17169 [M − H]^−^	351.12991 171.06636, 127.07649 ^a^, 123.04526, 99.08160	[15]
**55**	14.440	militarine	C_34_H_46_O_17_	726.27387	0.51	725.26599 [M − H]^−^	457.17157 ^a^, 285.09799, 153.05573, 127.07654, 123.04519	[17]
**Stilbenes**								
**38**	11.995	isorhapontigenin	C_15_H_14_O_4_	258.08932	−0.42	259.09647 [M + H]^+^	227.07019,199.07533 ^a^, 135.04410, 107.04953	[18]
**39**	12.018	rhaponticin	C_21_H_24_O_9_	420.14210	−0.16	419.13513 [M − H]^−^	256.07437, 241.05089 ^a^, 213.05588	[19]
**40**	12.116	piceatannol	C_14_H_12_O_4_	244.07371	−0.57	243.06630 [M − H]^−^	149.02441 ^a^, 121.02955, 93.03458	[20]
**57**	14.568	dihydro-resveratroll	C_14_H_14_O_3_	230.09433	−0.05	229.14445 [M − H]^−^	123.04518, 121.02949 ^a^ 107.05019, 93.03454	[21]
**64**	17.405	batatasin III	C_15_ H_16_O_3_	244.11001	0.23	245.11731 [M − H]^−^	227.10683, 151.07535, 137.05969, 121.06501 ^a^	[22]
**69**	19.445	3,3′-dihydroxy-4-(4-hydroxybenzyl)-5-methoxybibenzyl	C_22_H_22_O_4_	350.15206	0.71	349.14474 [M − H]^−^	255.10283, 243.10271 ^a^, 227.07153, 106.04240, 93.03458	[23]
**72**	19.998	bulbocodin C	C_29_H_28_O_5_	456.19405	0.83	455.18674 [M − H]^−^	361.14493 ^a^, 331.09796, 304.11102, 255.10280, 93.03461	[24]
**73**	20.542	bulbocodin D	C_29_H_28_O_5_	456.19372	0.88	455.18680 [M − H]^−^	440.09048, 361.1088 ^a^, 349.10840, 255.06645, 93.03416	[24]
**76**	22.298	3,3′-dihydroxy-2,6-bis(4-hydroxybenzyl)-5-methoxybibenzyl	C_29_H_28_O_4_	440.19894	0.42	439.19168 [M − H]^−^	424.16870, 345.14984 ^a^, 333.11353, 93.03459	[25]
**Phenanthrenes**								
**71**	19.863	1-((4-hydroxyphenyl)methyl)-4-methoxy-2,7-phenanthrenediol	C_22_H_18_O_4_	346.12087	1.03	347.12778 [M + H]^+^	253.08589 ^a^, 235.07544, 207.08047, 107.04955,	[26]
**74**	21.160	gymconopin A	C_22_H_20_O_4_	348.13616	0.02	347.12888 [M − H]^−^	332.10544 ^a^, 239.07147, 226.06348, 93.03457	[26]
**75**	21.191	9,10-dihydro-2-methoxy-4,5-phenanthrenediol	C_15_H_14_O_3_	242.09439	0.25	243.10161 [M + H]^+^	228.07809, 225.09105 ^a^, 211.07533 197.09607	[26]
**82**	26.152	blestriarene A	C_30_H_26_O_6_	482.17309	0.03	481.16586 [M − H]^−^	466.14246, 241.05086 ^a^, 210.06853	[26]
**83**	26.438	gymconopin	C_30_H_26_O_6_	482.17308	0.27	481.16583 [M − H]^−^	241.05081,225.09227, 210.06870 ^a^	[26]
**84**	27.870	blestriarene B	C_30_H_24_O_6_	480.15759	0.63	481.16461 [M + H]^+^	257.08075 ^a^, 225.05467, 211.07530, 207.04405	[26]
**Phenolic Acid Derivatives**								
**7**	4.203	(−)-4-[β-d-glucopyranosyl-(1-4)-β-d-glucopyranosyloxy]benzyl alcohol]	C_19_H_28_O_12_	448.15814	0.15	447.15176 [M − H]^−^	341.10901 ^a^,179.05614, 161.04562, 119.03497, 89.02443	[5]
**11**	4.877	(+)-4-[α-d-glucopyranosyl-(1-4)-β-d-glucopyranosyloxy]benzyl alcohol	C_19_H_28_O_12_	448.15811	0.12	447.15079 [M − H]^−^	341.10901 ^a^,179.05614, 161.04575, 89.02444, 71.01380	[5]
**13**	7.711	4-methoxyphenyl β-d-glucopyranoside	C_13_H_18_O_7_	286.10521	−0.16	285.09793 [M − H]^−^	179.11877, 161.04642, 123.04515 ^a^	[27]
**17**	8.943	dactylose B	C_12_H_16_O_6_	256.09481	0.49	255.08772 [M − H]^−^	237.11345,237.07713, 165.05467, 123.04523 ^a^	[28]
**18**	9.049	phenyl-3-deoxyheopyranoside	C_12_H_16_O_5_	240.09993	−0.63	239.09271 [M − H]^−^	179.07149 ^a^, 162.06873, 121.02957	[29]
**21**	9.267	isoferulic acid	C_10_H_10_O_4_	194.05803	0.64	195.06535 [M + H]^+^	177.05464 ^a^, 149.05975, 145.02840, 117.03376	[30]
**22**	9.549	ferulic acid	C_10_H_10_O_4_	194.05808	−0.88	195.06541 [M − H]^−^	177.05453, 149.05968, 145.02832 ^a^, 117.03370	[31]
**23**	9.562	*p*-doumaric acid	C_9_H_8_O_3_	164.04738	−0.23	163.04010 [M − H]^−^	119.05019 ^a^, 93.03452	[30]
**25**	9.621	(*E*)-4-methoxycinnamic acid	C_10_H_10_O_3_	178.06311	−0.69	179.07040 [M + H]^+^	147.04402 ^a^, 137.05974, 119.04941, 91.05477	[31]
**34**	11.595	tremuloidin	C_20_H_22_O_8_	390.13185	−0.97	389.12460 [M + H]^+^	341.10324, 193.05069 ^a^, 150.03229, 134.03743	[32]
**43**	12.631	chlorogenic acid	C_16_H_18_O_9_	354.09569	1.67	353.08841 [M − H]^−^	179.03511 ^a^,135.04527, 177.01929, 109.02952	[33]
**45**	13.353	quercetin-3β-D-glucoside	C_21_H_20_O_12_	464.09555	−0.15	463.08832 [M − H]^−^	300.02747 ^a^, 271.02481, 255.02997	[34]
**49**	13.665	cirsimarin	C_23_H_24_O_11_	476.13197	−0.22	475.12469 [M − H]^−^	307.08240 ^a^, 167.03502, 152.01154	[35]
**50**	14.041	astragalin	C_21_H_20_O_11_	448.10073	−0.39	447.09341 [M − H]^−^	284.03262, 255.03510 ^a^, 227.03510	[36]
**56**	14.470	kaempferol-7-*O*-glucoside	C_21_H_20_O_11_	448.10072	−0.36	449.10794 [M + H]^+^	287.05487 ^a^, 258.05228, 145.04948	[37]
**59**	14.609	desmethylxanthohumol	C_18_H_22_O_5_	340.13105	0.07	341.13831 [M + H]^+^	323.12762, 217.08611, 153.05446, 137.05969 ^a^, 187.07526	[38]
**61**	14.917	isorhamnetin	C_16_H_12_O_7_	316.05854	−0.74	317.06573 [M + H]^+^	302.04196 ^a^, 274.04684, 273.03922, 153.01820	[39]
**63**	16.015	naringenin chalcone	C_15_H_12_O_5_	272.06856	−0.33	271.06131 [M − H]^−^	177.01930, 151.00363 ^a^, 145.02951, 119.05019	[40]
**65**	17.450	equol	C_15_H_14_O_3_	242.09429	−0.72	243.10172 [M − H]^−^	228.07822, 211.07527, 149.05972, 135.04405, 123.04429,107.04951 ^a^	[41]
**82**	24.670	galangin	C_15_H_10_O_5_	270.05291	−0.31	269.04562 [M − H]^−^	241.05077, 225.05580 ^a^	[42]
**Alkaloids**								
**1**	1.112	dl-arginine	C_6_H_14_N_4_O_2_	174.11176	−0.48	175.11899 [M + H]^+^	158.09248,130.09763,116.07089, 112.08723, 70.06586 ^a^	[43]
**3**	1.946	Adenosine	C_10_H_13_N_5_O_4_	267.09653	0.84	268.10388 [M + H]^+^	136.06180^a^, 119.03542,	[43]
**4**	1.961	6-quinolinecarboxylic acid	C_10_ H_7_NO_2_	173.04785	0.03	174.05510 [M + H]^+^	156.04442, 146.06017 ^a^, 130.06531,128.04971	[44]
**5**	2.479	l-Phenylalanine	C_9_H_11_NO_2_	165.07921	−1.40	166.08640 [M + H]^+^	149.05977, 131.04926, 120.08099 ^a^,103.05462	[45]
**6**	3.100	*N*-(4-methyoxyphenyl)-1H-pyrazolo [3,4-d]pyrimidin	C_12_H_11_N_5_O	241.09636	−0.14	242.10341 [M + H]^+^	136.06171, 107.04944 ^a^	[46]
**8**	4.329	*trans*-indole-3-acrylic acid	C_11_H_9_NO_2_	187.06348	−0.29	188.07060 [M + H]^+^	170.06012, 146.06004 ^a^, 144.08080, 118.06541	[47]
**10**	4.856	Guanine	C_5_H_5_N_5_O	151.04946	−0.34	152.05661 [M + H]^+^	135.03011 ^a^, 110.03517	[48]
**12**	5.444	5′-S-Methyl-5′-thioadenosine	C_11_H_15_N_5_O_3_S	297.08965	−0.29	298.09668 [M + H]^+^	136.06178 ^a^, 163.04239, 145.03169	[49]
**14**	8.361	conopsamide A	C_14_H_21_N_3_O_4_	295.15315	1.05	294.14621 [M − H]^−^	188.10416, 131.08266 ^a^,	[50]
**15**	8.420	befunolol	C_16_H_21_NO_4_	291.14681	0.90	292.25405 [M + H]^+^	277.13074, 151.03897, 124.11227 ^a^,	[51]
**19**	9.067	cyclo(tyrosy-tyrosyl)	C_18_H_18_N_2_O_4_	326.12667	−0.05	327.13342 [M + H]^+^	221.09201, 203.08133, 175.08655,158.06003, 107.04946 ^a^	[6]
**24**	9.596	cyclo(leucylprolyl)	C_11_H_18_N_2_O_2_	210.13695	0.58	211.14403 [M + H]^+^	193.08359, 183.14925, 138.12781, 127.08688, 114.09170, 70.06586 ^a^	[52]
**26**	9.758	*N*-(4-hydroxybenzy) adenine riboside	C_17_H_19_N_5_O_5_	373.13861	−0.05	374.14581 [M + H]^+^	242.10358, 148.06180, 136.06180 ^a^, 107.04951	[53]
**27**	9.827	dibenzylamine	C_14_H_15_N	197.12062	−0.89	198.12784 [M + H]^+^	181.10126, 106.06558,91.05482 ^a^	[54]
**30**	10.699	(+)-chelidonine	C_20_H_19_NO_5_	353.12643	−0.30	354.13321 [M + H]^+^	336.12274,293.08057, 188.07043 ^a^, 206.08098, 149.05965	[55]
**37**	11.822	(2E)-3-(4-hydroxy-phenyl)-*N*-[2-(4-hydroxy-phenyl)-ethyl]-acrylamide	C_17_H_17_NO_3_	283.12083	0.06	284.12769 [M + H]^+^	147.04390 ^a^, 164.07062, 121.06493, 119.04931	[56]
**42**	12.834	2,3,4,9-tetrahydro-1H-β-carboline-3-carboxylic acid	C_12_H_12_N_2_O_2_	216.09012	−1.13	217.09723 [M + H]^+^	144.08080 ^a^, 156.08093, 118.06545	[57]
**58**	14.582	dl-tryptophan	C_11_H_12_N_2_O_2_	204.08987	0.03	203.08272 [M − H]^−^	159.09279, 142.06619, 116.05058 ^a^, 74.24770	[48]
**78**	23.937	*N*-phenyl-2-naphthylamine	C_16_H_13_N	219.10478	0.08	220.11194 [M + H]^+^	143.07289 ^a^, 128.06215	[58]
**Terpenoids and Steroids**								
**41**	12.664	mascaroside	C_26_H_36_O_11_	524.22615	−0.73	523.21875 [M − H]^−^	361.6602 ^a^, 179.07140, 165.05576, 101.02450	[59]
**51**	14.349	(±)-abscisic acid	C_15_H_20_O_4_	264.13613	0.12	263.12869 [M − H]^−^	219.13905 ^a^,204.11546, 201.12842, 151.07640	[60]
**77**	23.323	(3β,5α,9α)-3,6,19-trihydroxyurs-12-en-28-oic acid	C_30_H_48_O_5_	488.35032	−0.29	489.35718 [M + H]^+^	471.34665 ^a^,453.33636, 435.32520, 265.21689	[61]
**80**	24.638	(3β,17β)-estr-5(10)-ene-3,17-diol	C_18_H_28_O_2_	276.20882	0.12	277.21600 [M + H]^+^	259.20557, 235.16937, 221.15327, 149.13251, 121.10139, 107.08587, 93.07037 ^a^,	[62]
**85**	28.595	17α-methyl-5α-androstane-3β,11β,17β-triol	C_20_H_34_O_3_	322.25091	0.37	323.25797 [M + H]^+^	305.24716, 277.21613 ^a^, 259.20554, 179.14297, 151.11176, 135.11687, 107.08589	[63]
**86**	32.654	lup-20(29)-en-28-al	C_30_H_48_O_2_	440.36543	−0.04	441.37292 [M + H]^+^	423.36244 ^a^, 405.35190, 191.14313, 151.11177, 109.10156, 123.08073	[64]
**87**	33.514	lupenone	C_30_H_48_O	424.37052	−0.02	425.37735 [M + H]^+^	407.36710 ^a^, 231.21080, 191.17928, 177.16399, 109.10153	[65]
**88**	34.104	poriferasterol	C_29_H_48_O	412.37052	−0.07	413.37762 [M + H]^+^	395.36703 ^a^,353.33051, 255.21051, 213.16359, 159.11682, 105.07026	[66]
**89**	35.684	4,4-dimethyl-5α-cholesta-8,14,24-trien-3β-ol	C_29_H_46_O	410.35496	−0.12	411.36194 [M + H]^+^	393.35141, 353.32016, 253.19467, 175.11179 ^a^, 147.11678	[67]
**90**	40.568	lupeol	C_30_H_50_O	426.38611	0.13	427.39322 [M + H]^+^	409.38208, 191.17934, 121.10136, 109.10149, 95.08600 ^a^	[68]
**91**	41.305	(22E)-stigmasta-3,5,22-triene	C_29_H_46_	394.35992	0.06	395.36719 [M + H]^+^	297.25775, 241.19502, 173.13257, 159.11693, 145.10123 ^a^	[69]
**Others**								
**2**	1.354	citric acid	C_6_H_8_O_7_	192.02699	0.05	191.01979 [M − H]^−^	173.00919, 129.01920, 111.00877 ^a^, 87.00876,	[70]
**20**	9.247	butanedioic acid	C_8_H_14_O_5_	190.08414	0.15	189.07680 [M − H]^−^	171.06630, 129.05573 ^a^, 143.07171, 127.07654, 99.08161	[71]
**60**	14.911	pinoresinol	C_20_H_22_O_6_	358.1417	0.75	359.14969 [M − H]^−^	163.03735, 137.05968 ^a^, 131.04922	[72]
**62**	15.501	benzyl-[(6-oxo-7,8,9,10-tetrahydro-6H-benzo[c]chromen-3yl)oxy]-acetate	C_22_H_20_O_5_	364.13133	−0.72	365.13849 [M + H]^+^	271.09637, 239.07021, 147.04408, 107.04951 ^a^	[72]
**66**	18.242	aloeresin A	C_28_H_28_O_11_	540.16377	−1.15	539.15643 [M − H]^−^	377.10330 ^a^, 283.06125, 163.00378	[73]
**67**	19.175	frangulin B	C_20_H_18_O_9_	402.09545	−0.9	401.08740 [M − H]^−^	357.06149, 313.07181, 121.02949 ^a^	[74]
**68**	19.422	cleomiscosin A	C_20_H_18_O_8_	386.10051	−0.91	387.10724 [M + H]^+^	357.06030 ^a^, 329.06540, 301.07065, 245.04463, 149.05989	[75]
**70**	19.772	bis-(methylbenzylidene)-sorbitol	C_22_H_26_O_6_	386.17321	−0.69	387.18051 [M + H]^+^	105.07003 ^a^, 119.04945, 103.05464	[75]
**80**	24.129	umbelliferone	C_9_H_6_O_3_	162.03168	0.09	163.03894 [M + H]^+^	135.04408 ^a^,133.02847, 107.04951, 105.04509	[33]

^a^ Basepeak.

## Supplementary Materials

The following Supplementary Materials are available online: The Figures S1–S42 showed the chemical structures and available raw MS2 spectra of some compounds identified from the tubers of *G. conopsea*.
