# Supplementary material for: Rapid Characterizaiton of Chemical Constituents of the Tubers of Gymnadenia conopsea by UPLC–Orbitrap–MS/MS Analysis
Source: Molecules. 2020 Feb 18;25(4):898. doi: 10.3390/molecules25040898 (PMC7070944; doi:10.3390/molecules25040898)
Supplement: Supplementary file 1 [file molecules-25-00898-s001.pdf]

Supplementary Matrials

Rapid Characterizaiton of Chemical Constituents of the tubers of *Gymnadenia conopsea* by UPLC-Orbitrap-MS/MS analysis

Xin Wang[1], Xian-Jian Zhong[1], Na Zhou[1], Ning Cai[1], Jia-Hui Xu[1], Qing-Bo Wang[1], Jin-Jie Li[1], Qian Liu[1], Peng-Cheng Lin[2] and Xiao-Ya Shang[1*]

1 Beijing Key Laboratory of Bioactive Substances and Functional Foods, Beijing Union University, Beijing 100191, China

2 Qinhai Provincial Key Laboratory of Phytochemistry for Tibetan Plateau, Qinghai University for Nationalities, Xining 810000, China

* Correspondence: shangxiaoya@buu.edu.cn; Tel.: +86-010-6200-4533

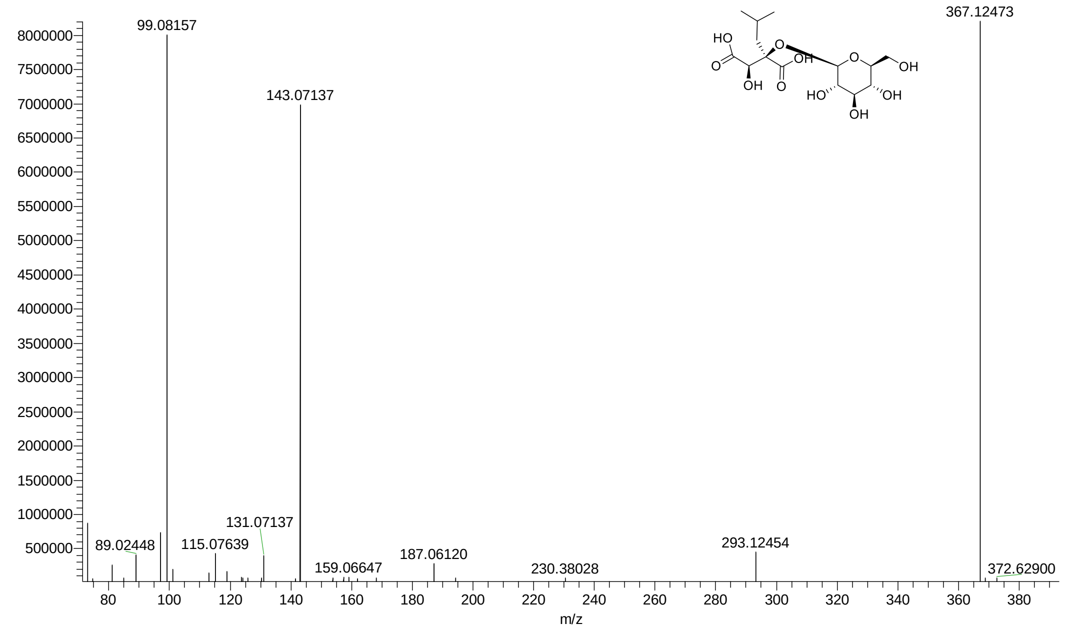

Figure S1. The MS/MS2 spectrum of compound **9.**

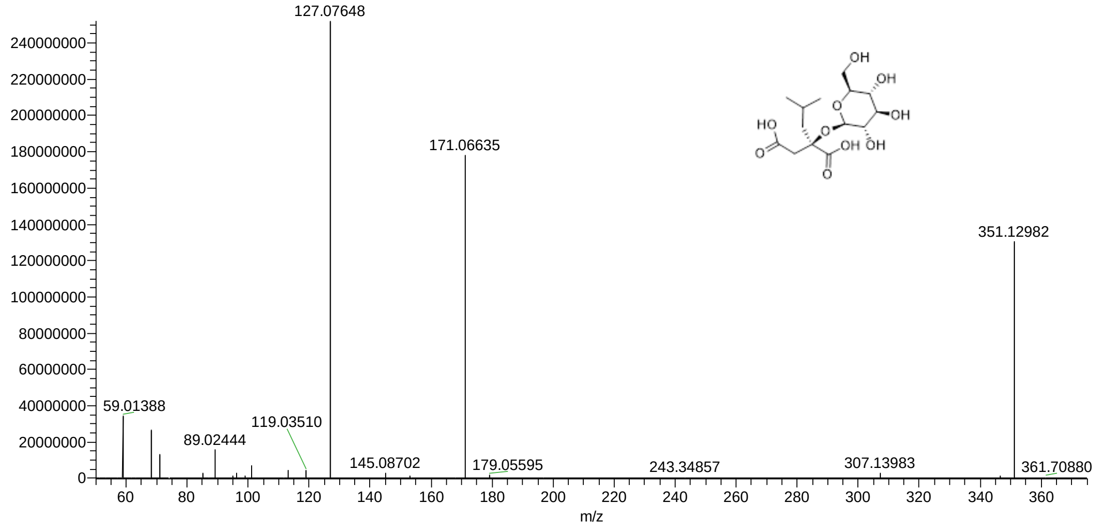

Figure S2. The MS/MS2 spectrum of compound **16.**

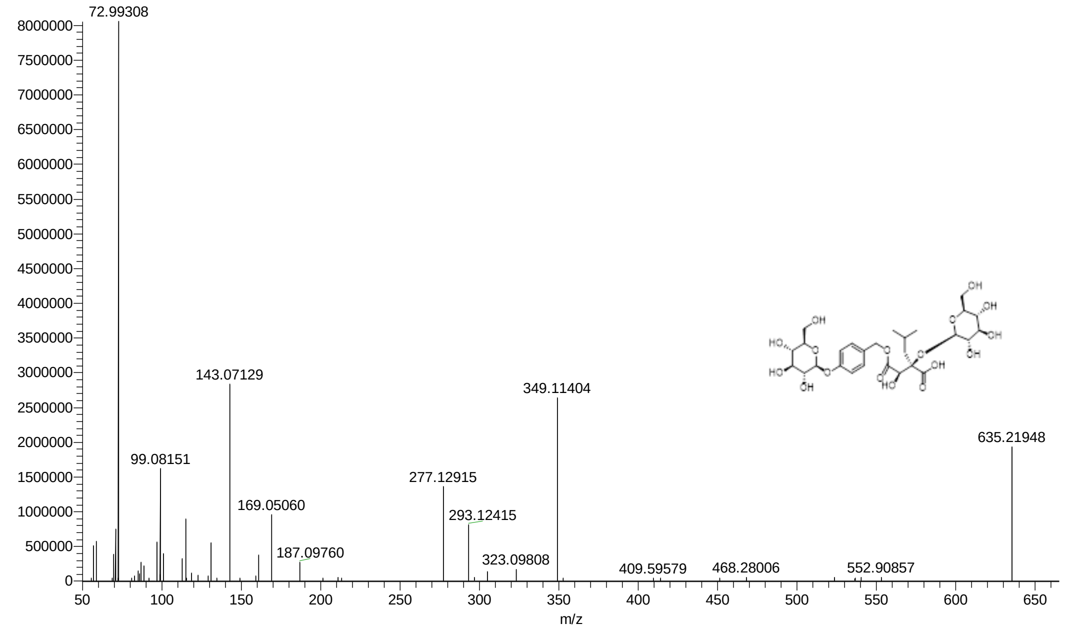

Figure S3. The MS/MS2 spectrum of compound **28.**

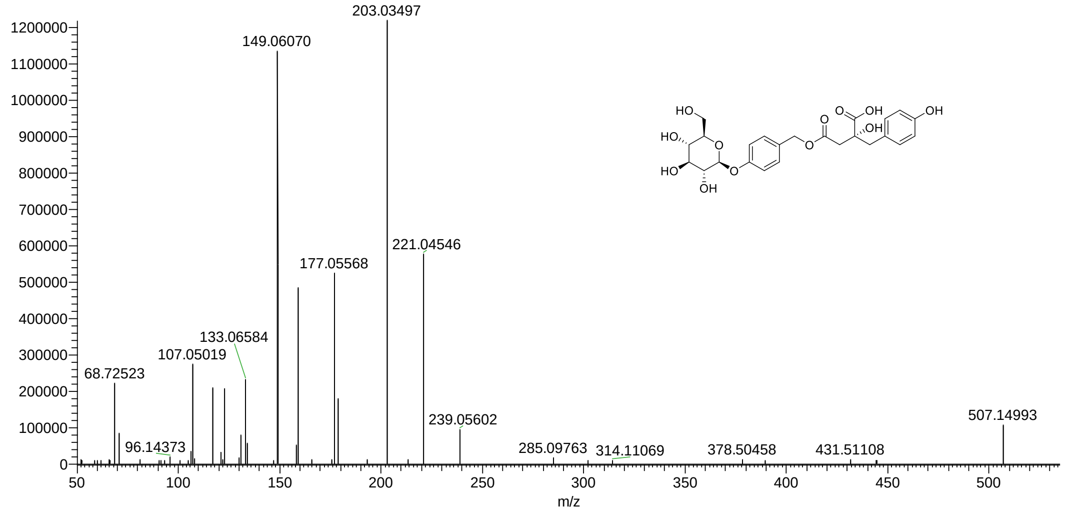

Figure S4. The MS/MS2 spectrum of compound **29.**

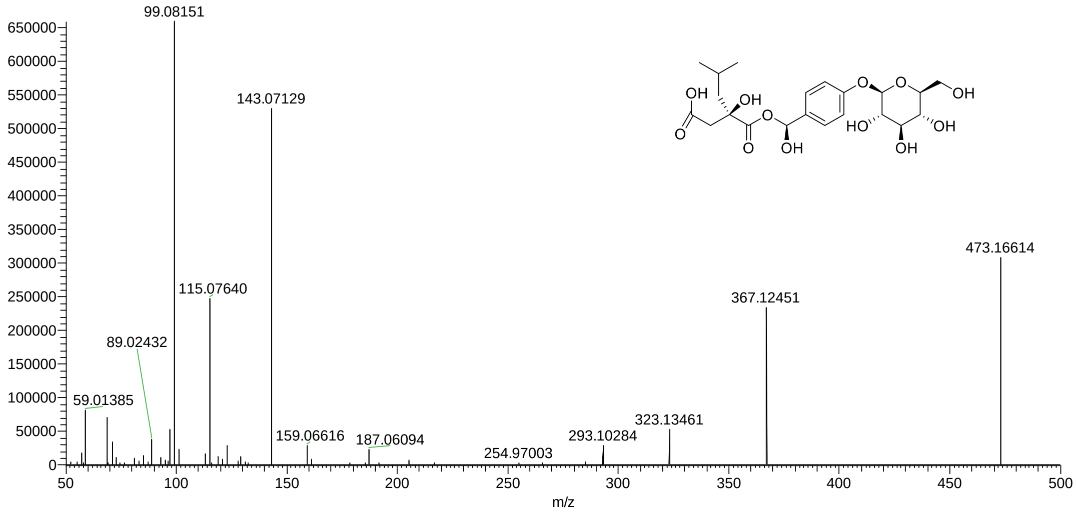

Figure S5. The MS/MS2 spectrum of compound **31.**

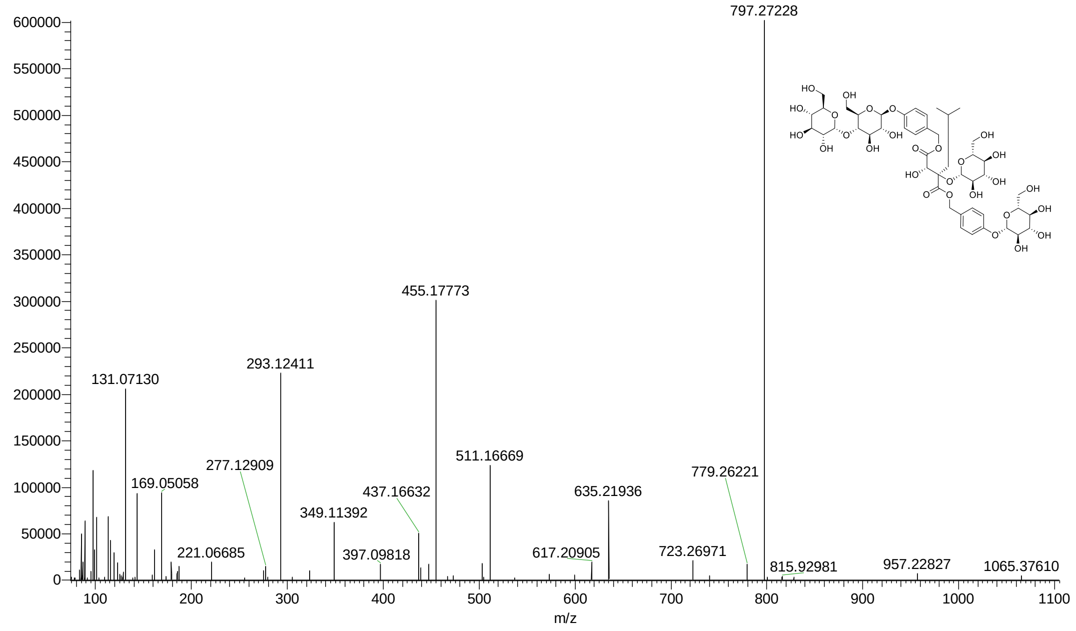

Figure S6. The MS/MS2 spectrum of compound **32.**

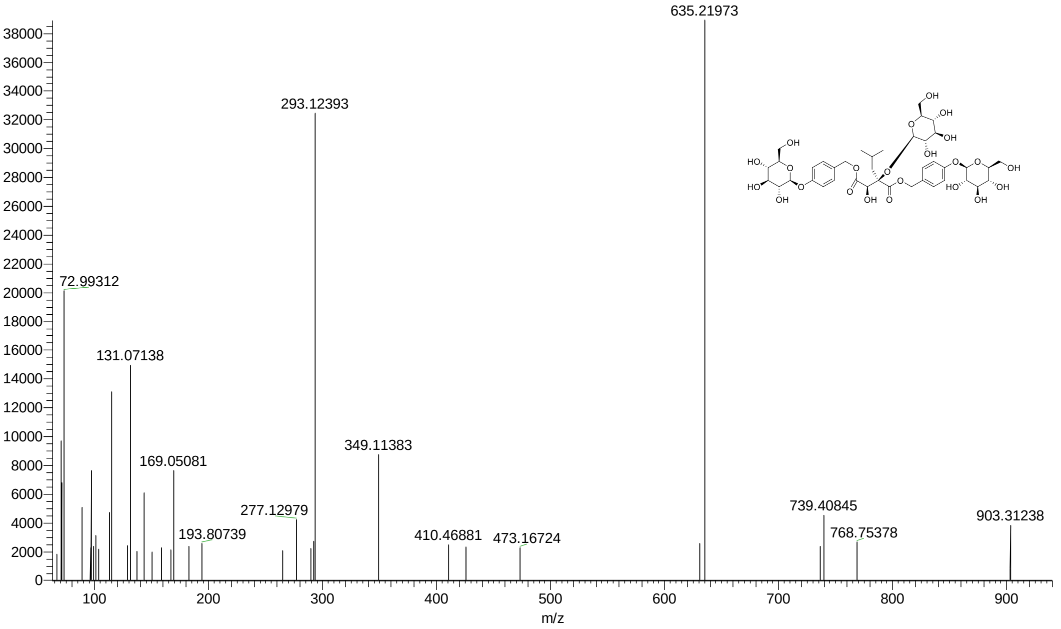

Figure S7. The MS/MS2 spectrum of compound **37.**

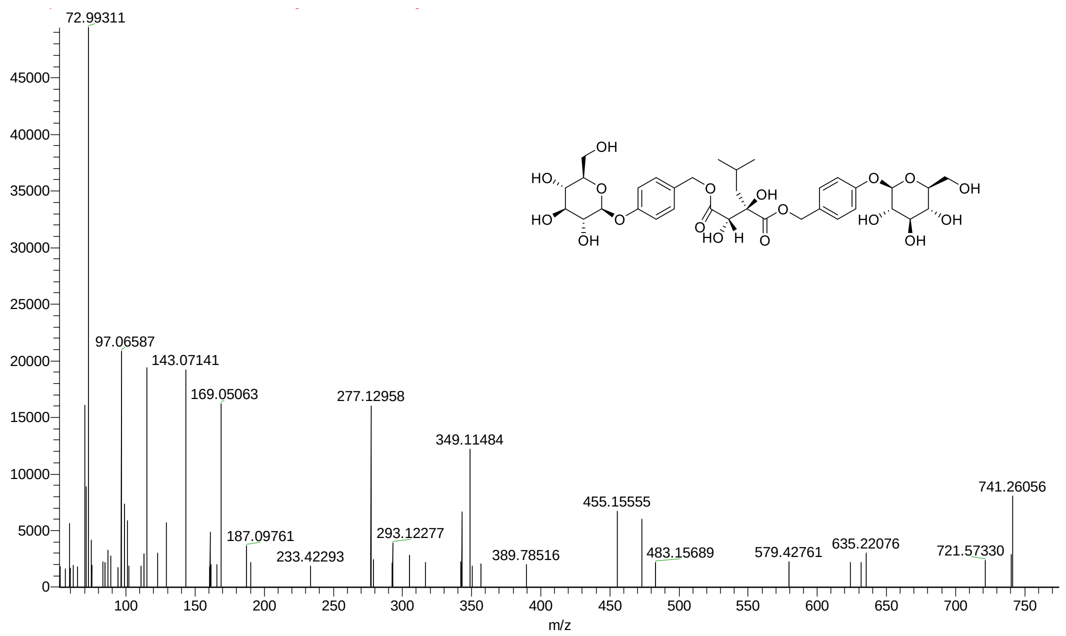

Figure S8. The MS/MS2 spectrum of compound **35.**

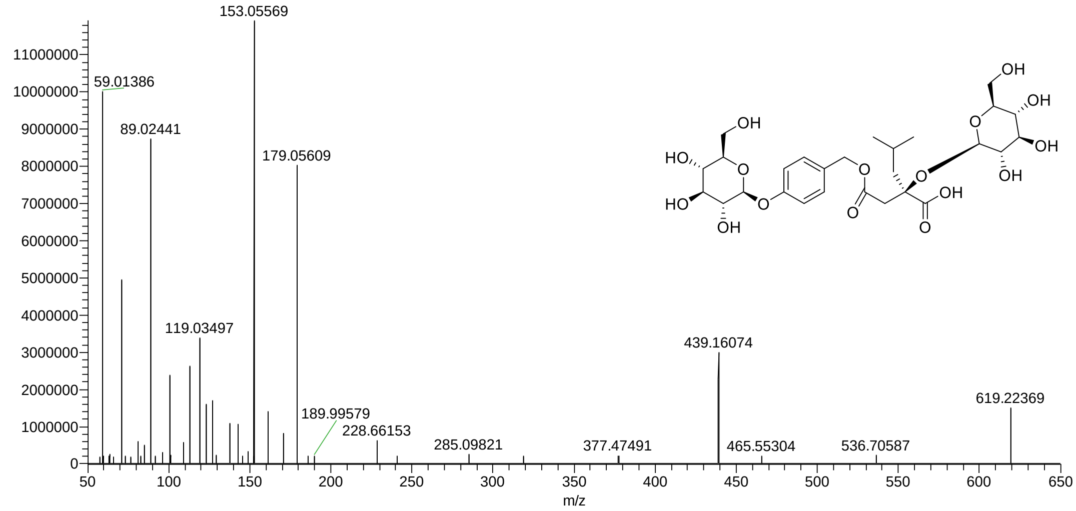

Figure S9. The MS/MS2 spectrum of compound **36.**

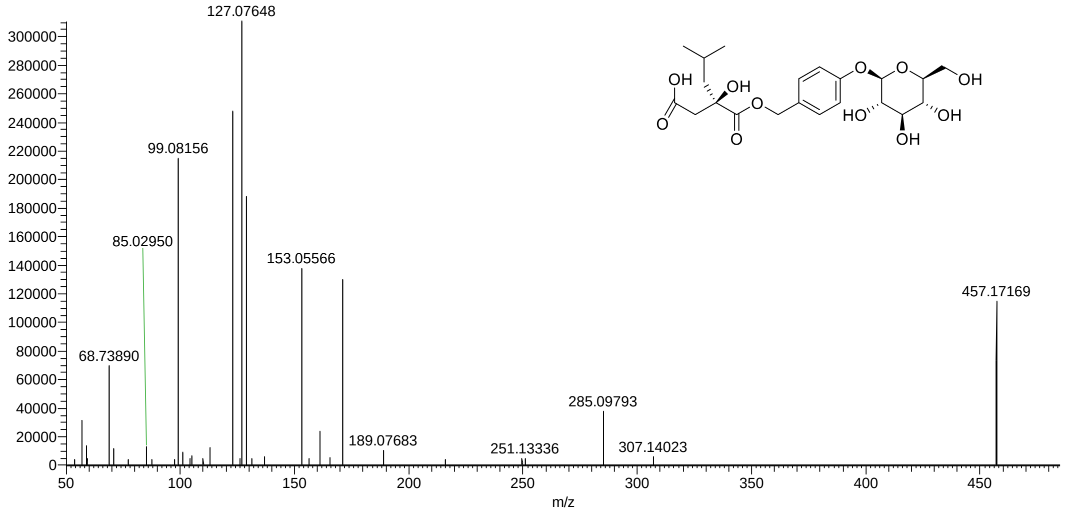

Figure S10. The MS/MS2 spectrum of compound **44.**

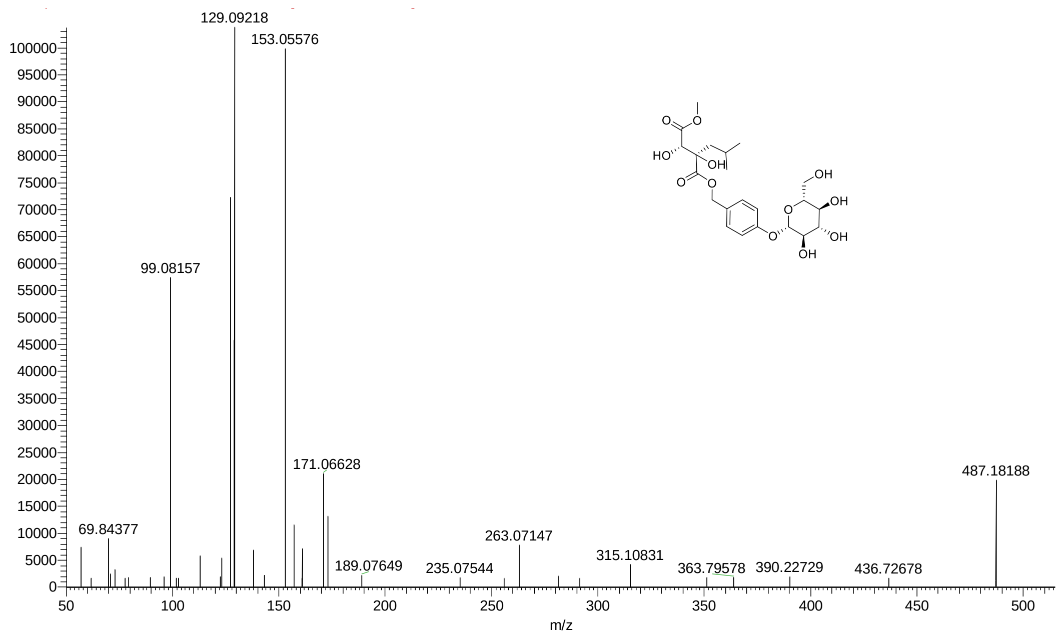

Figure S11. The MS/MS2 spectrum of compound **46.**

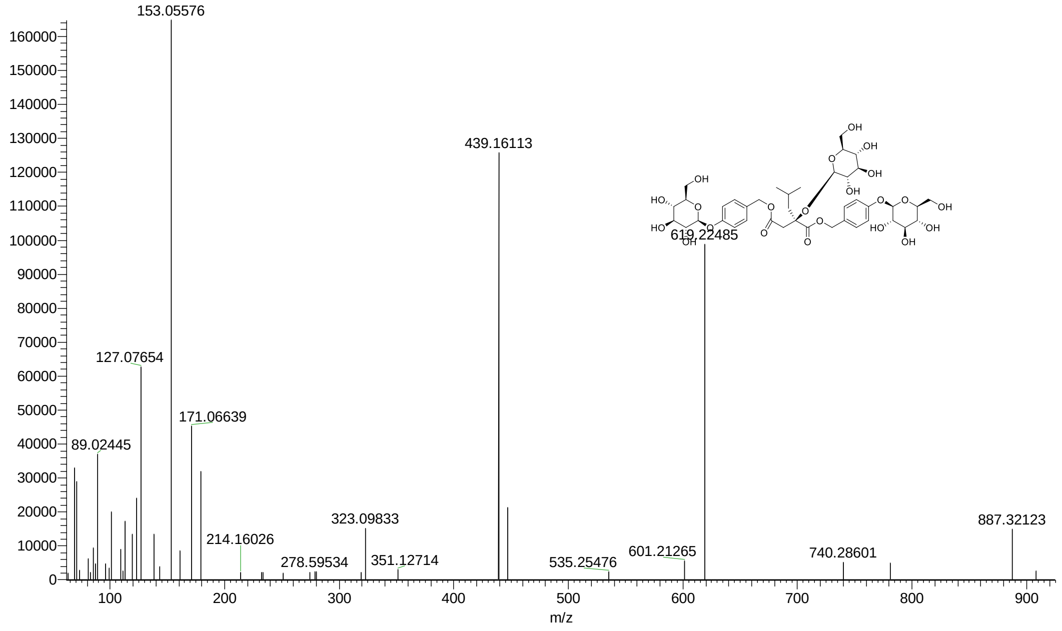

Figure S12. The MS/MS2 spectrum of compound **47.**

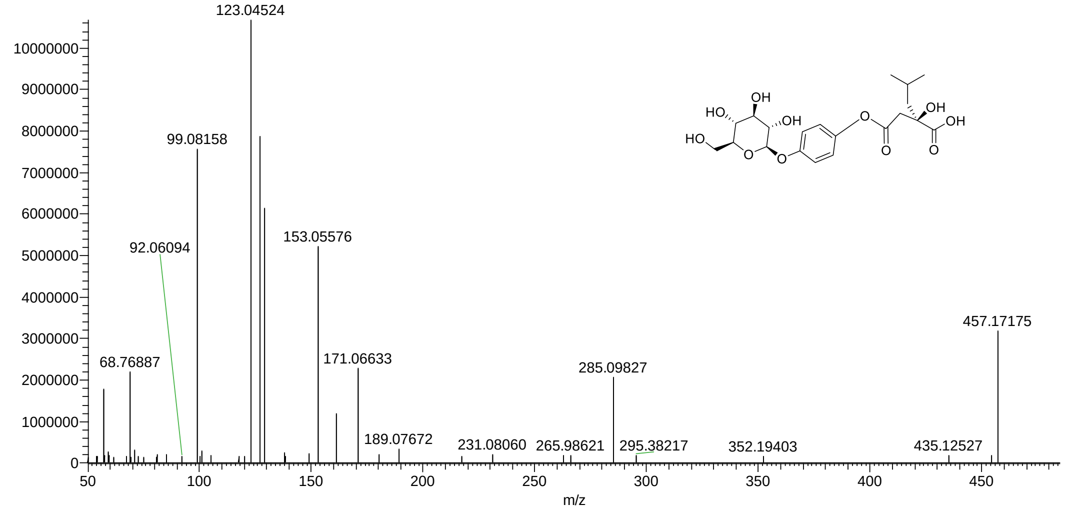

Figure S13. The MS/MS2 spectrum of compound **48.**

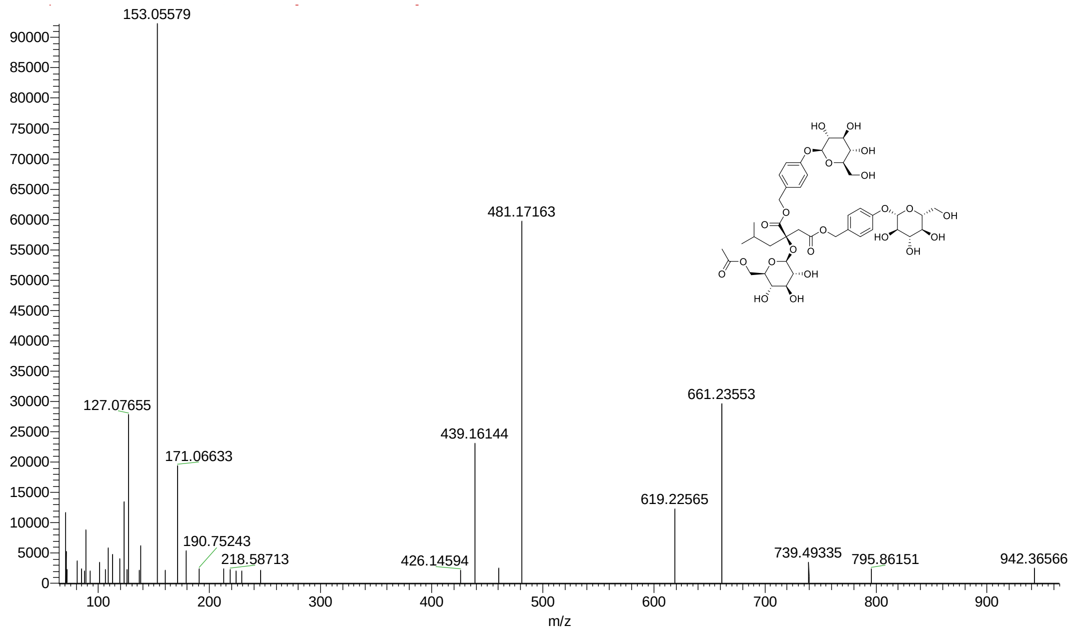

Figure S14. The MS/MS2 spectrum of compound **52.**

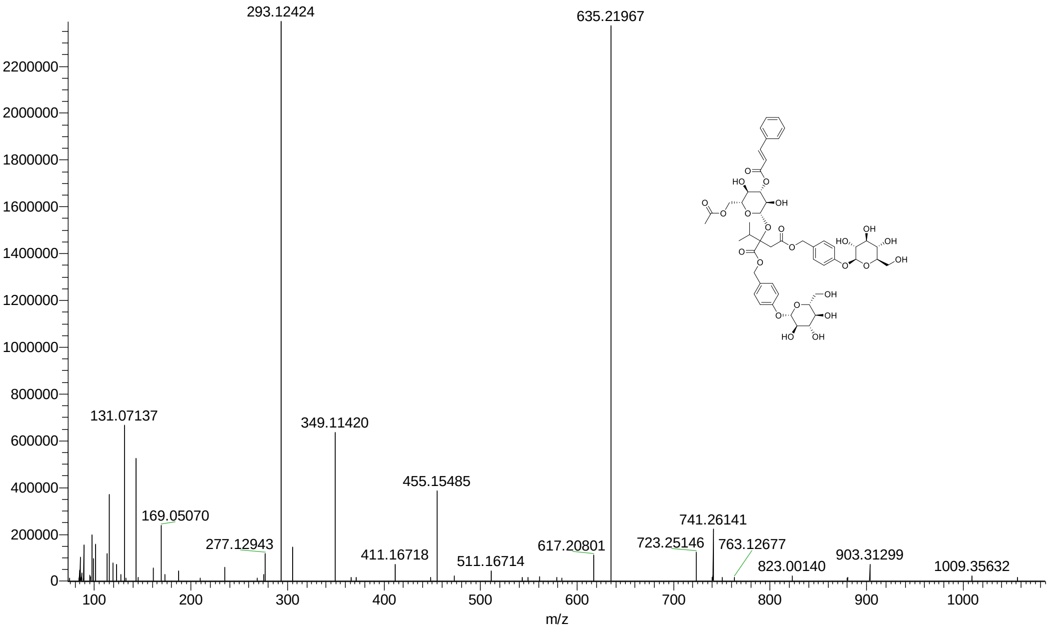

Figure S15. The MS/MS2 spectrum of compound **53.**

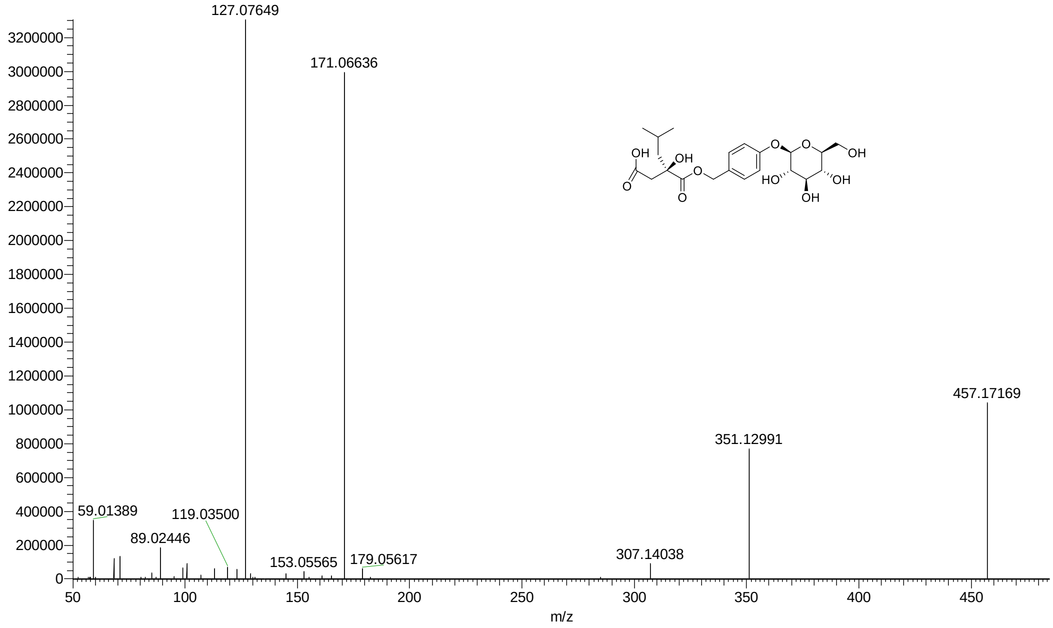

Figure S16. The MS/MS2 spectrum of compound **54.**

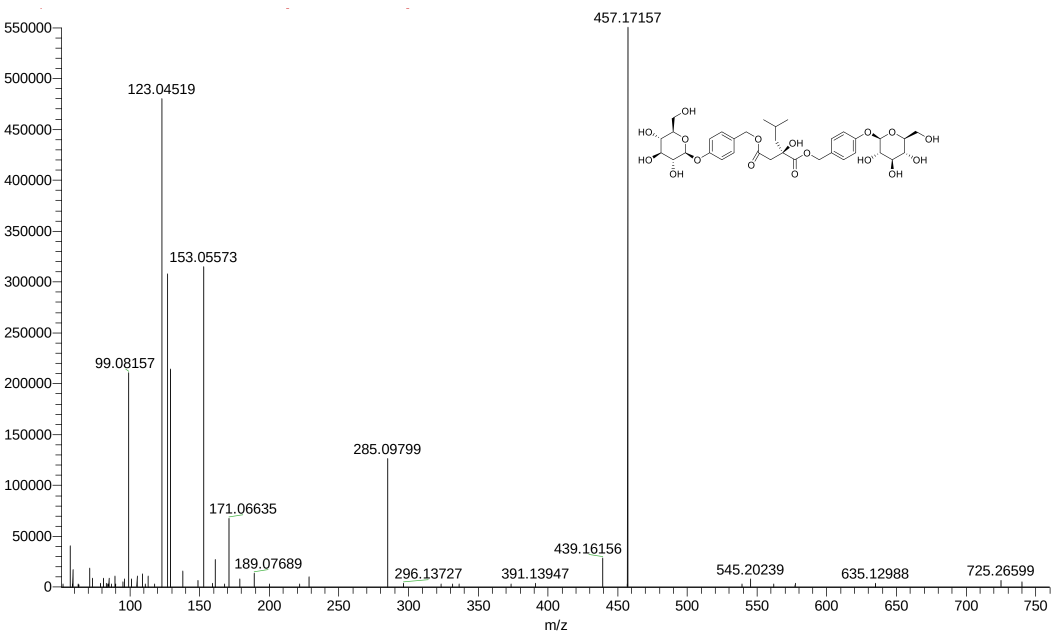

Figure S17. The MS/MS2 spectrum of compound **55.**

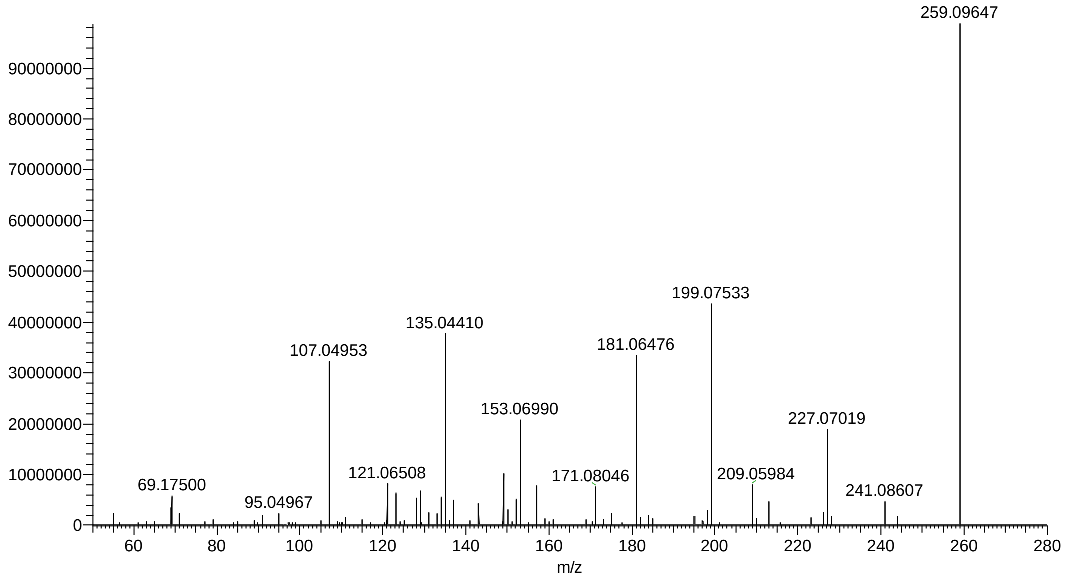

Figure S18. The MS/MS2 spectrum of compound **38.**

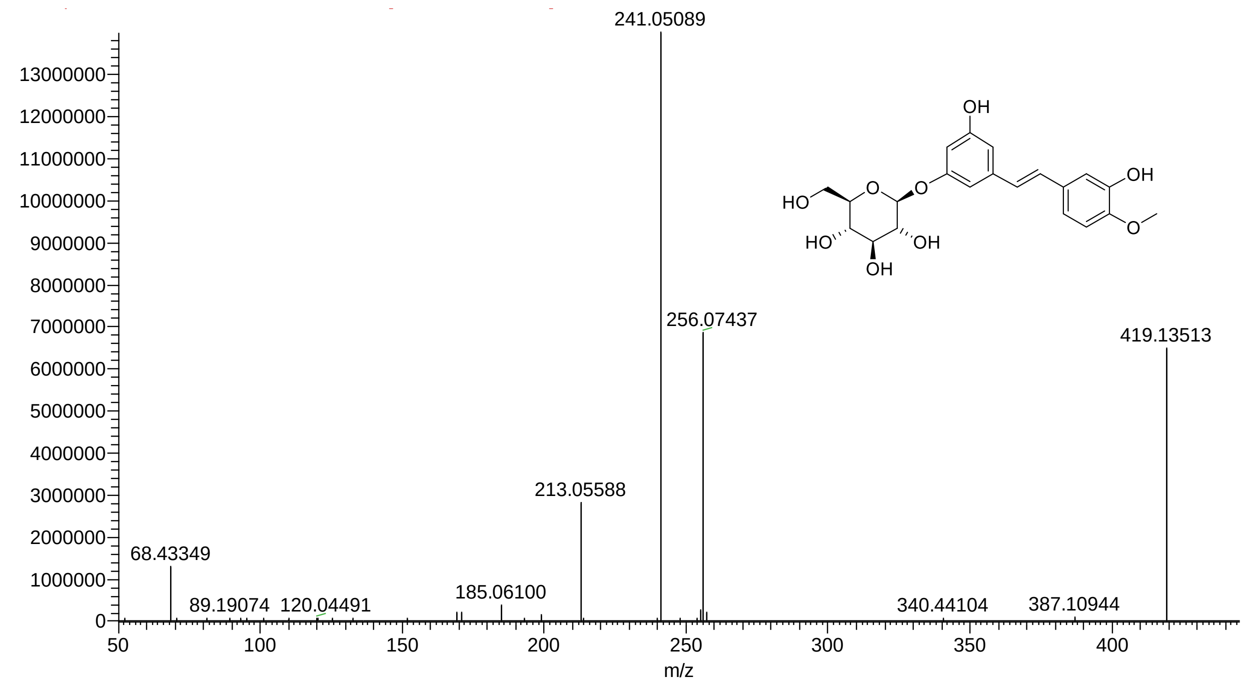

Figure S19. The MS/MS2 spectrum of compound **39.**

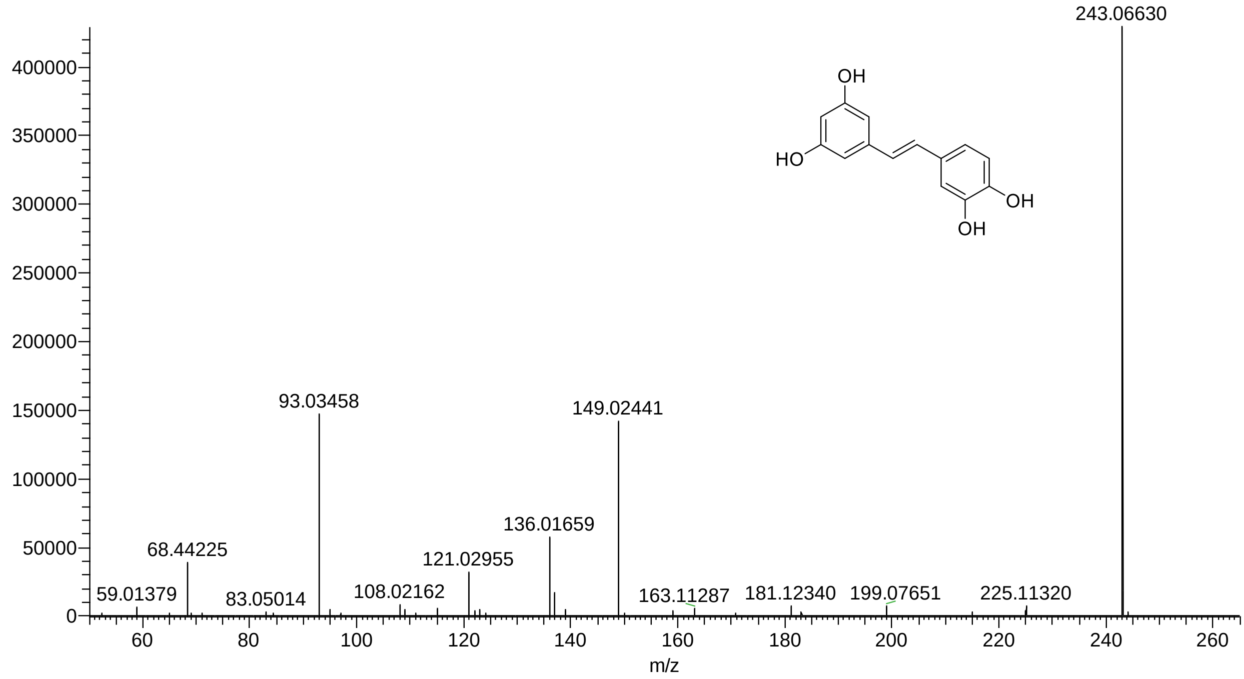

Figure S20. The MS/MS2 spectrum of compound **40.**

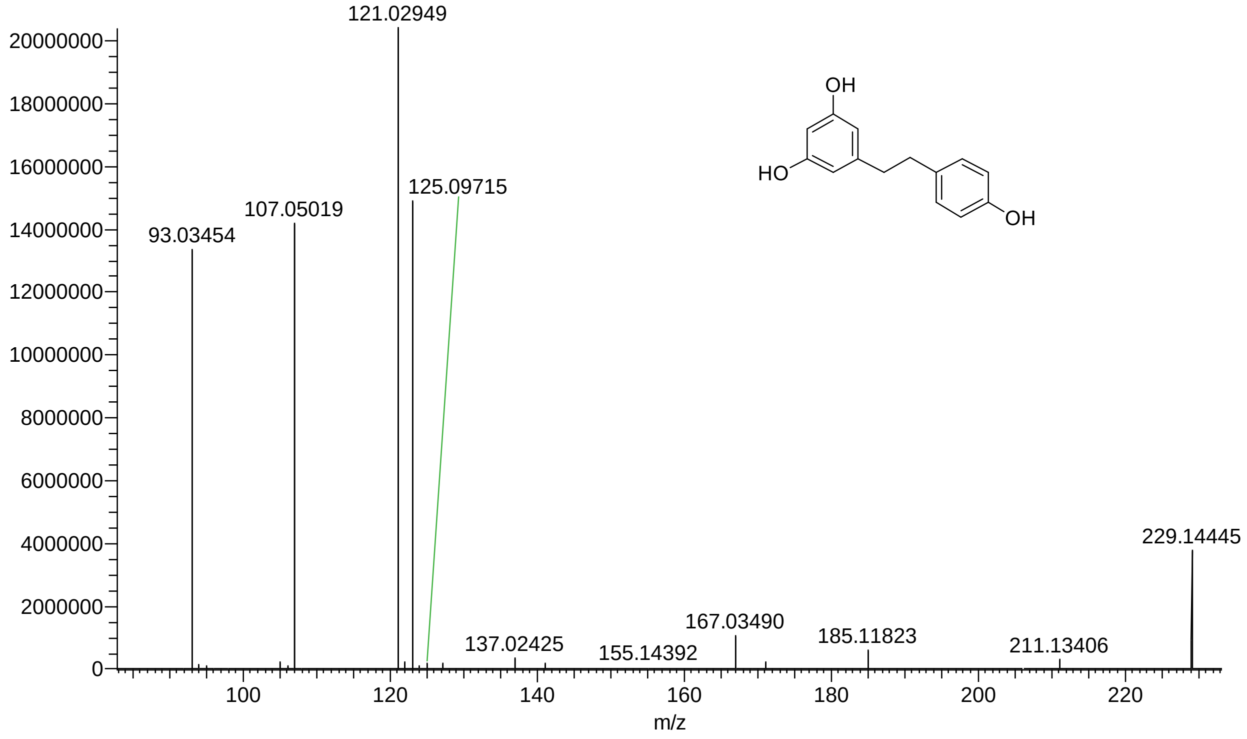

Figure S21. The MS/MS2 spectrum of compound **57.**

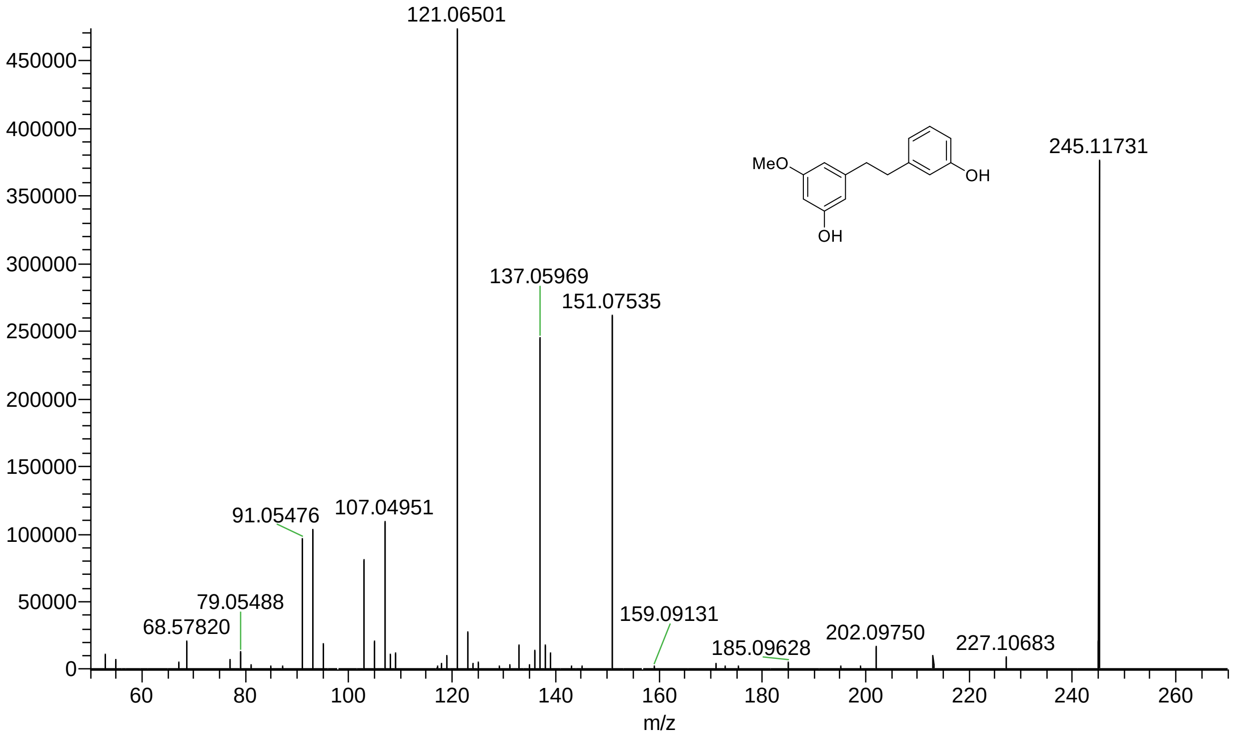

Figure S22. The MS/MS2 spectrum of compound **64.**

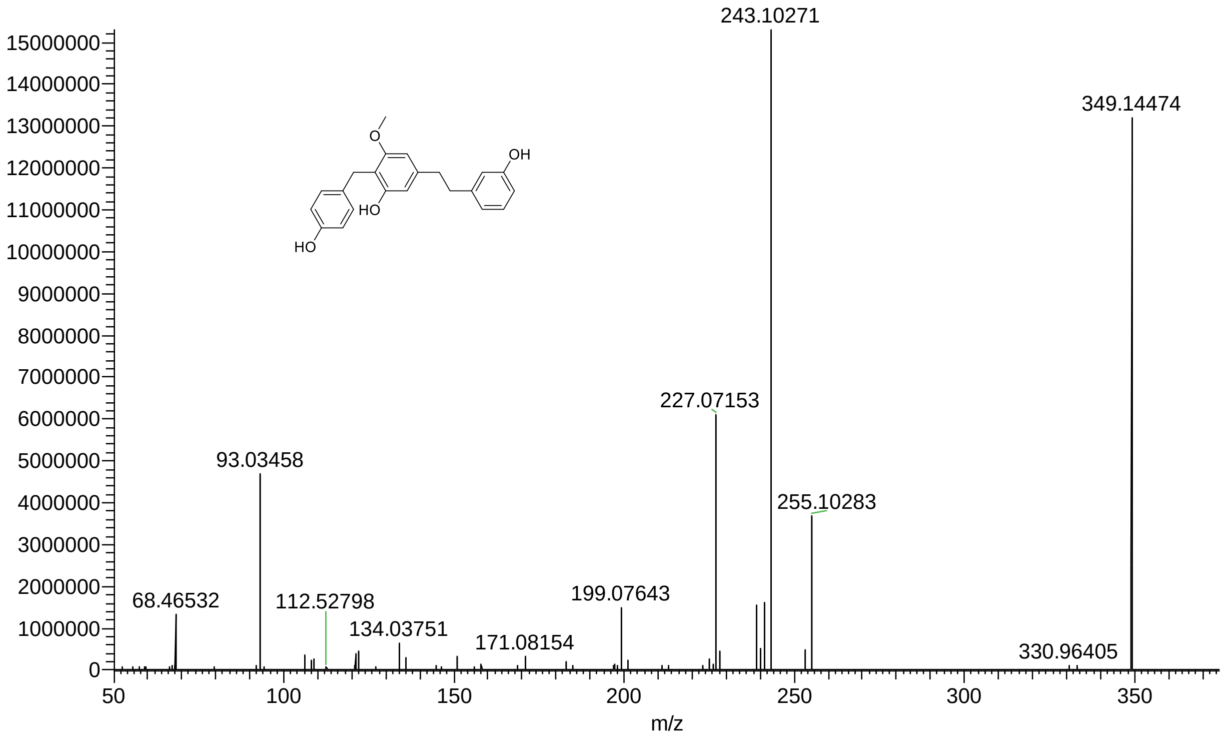

Figure S23. The MS/MS2 spectrum of compound **69.**

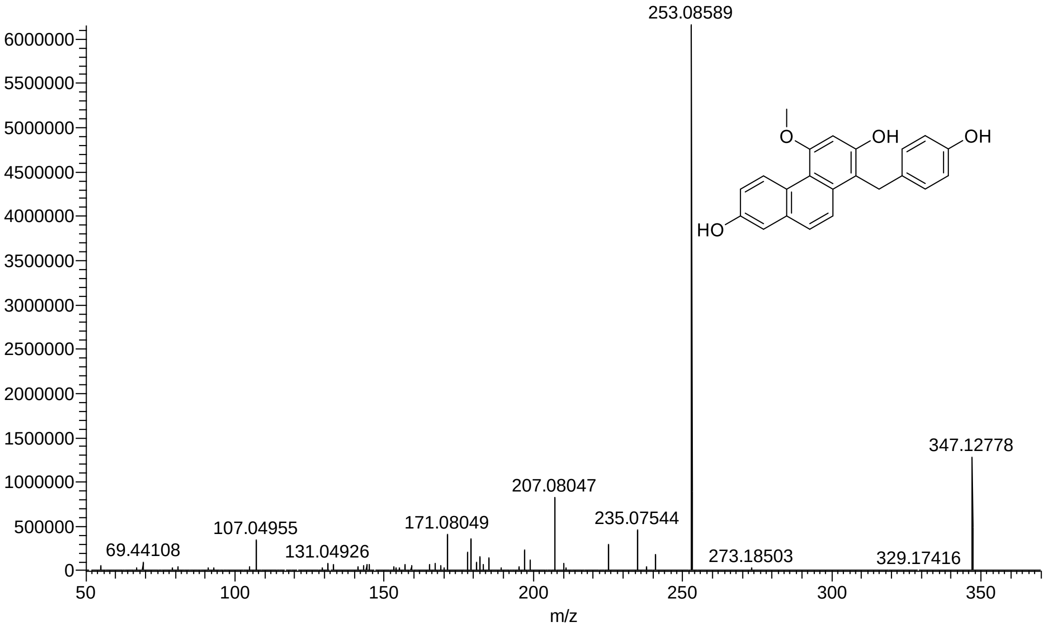

Figure S24. The MS/MS2 spectrum of compound **69.**

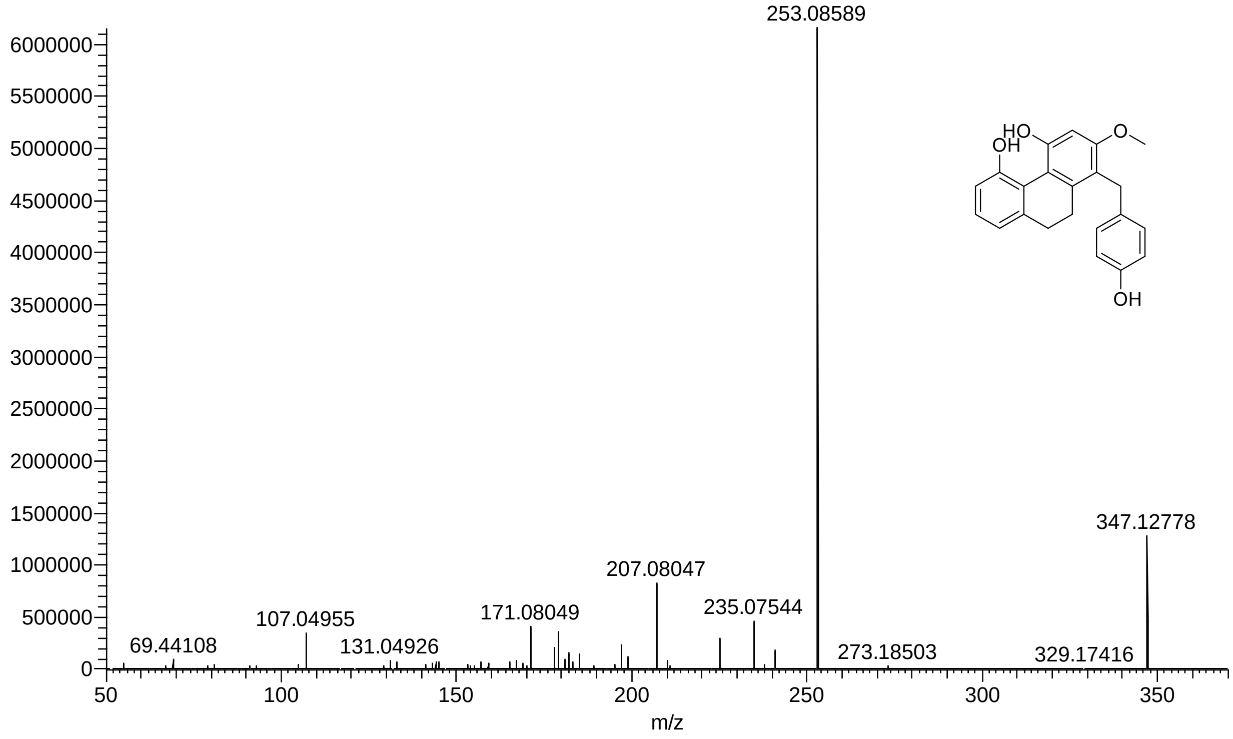

Figure S25. The MS/MS2 spectrum of compound **71.**

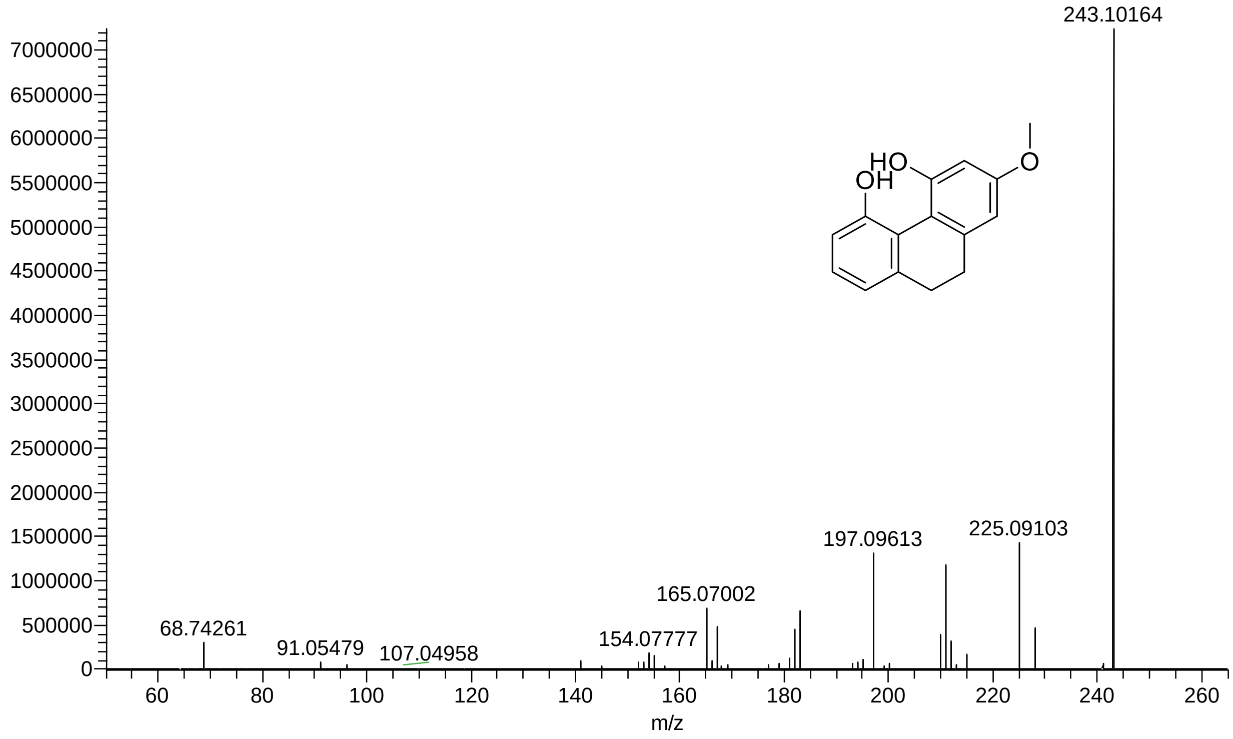

Figure S26. The MS/MS2 spectrum of compound **75.**

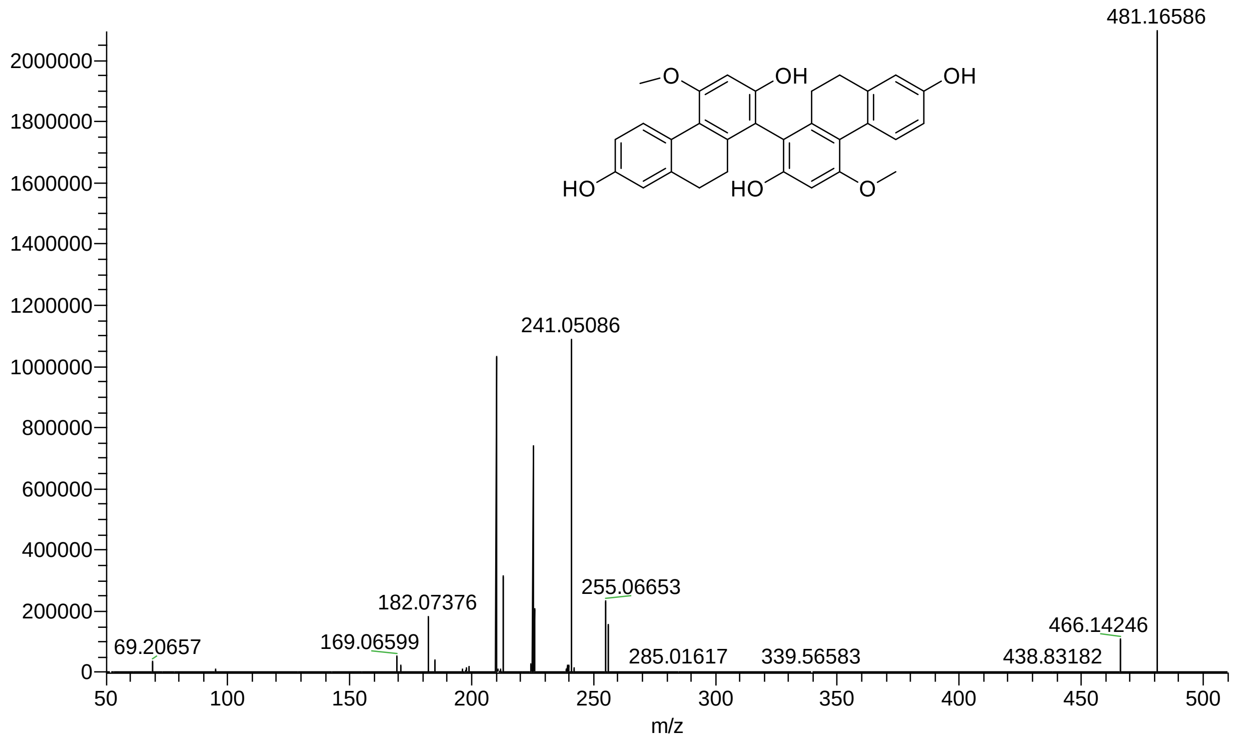

Figure S27. The MS/MS2 spectrum of compound **82.**

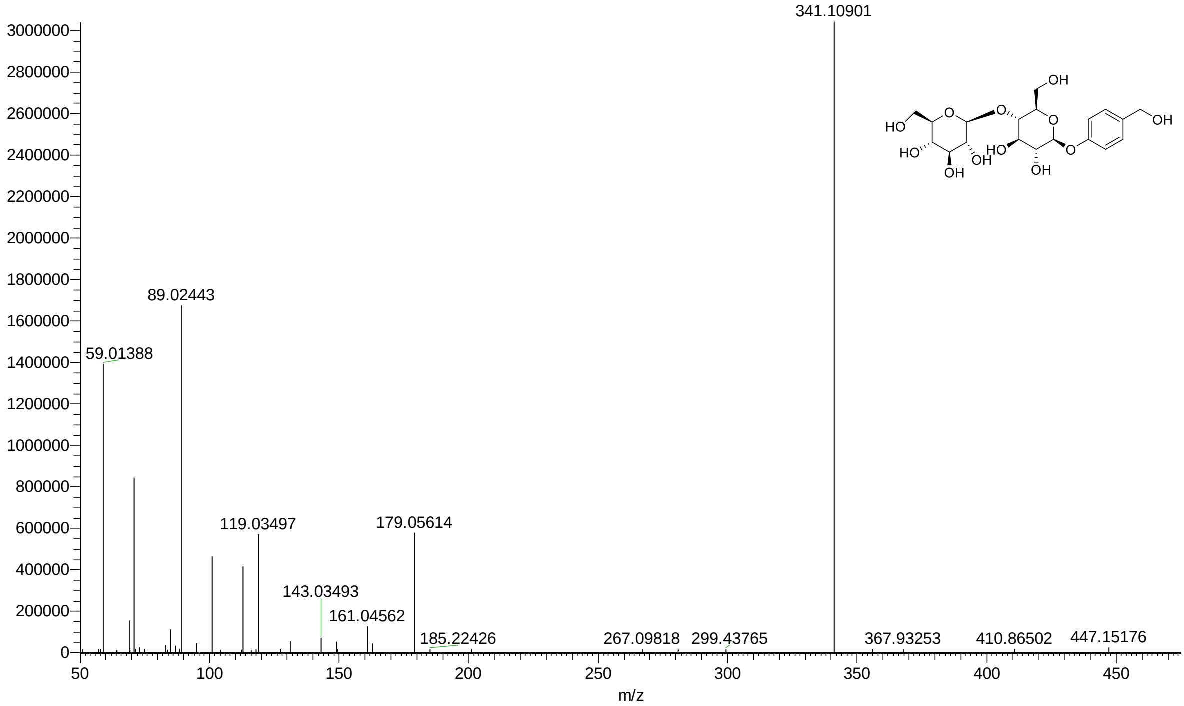

Figure S28. The MS/MS2 spectrum of compound **7.**

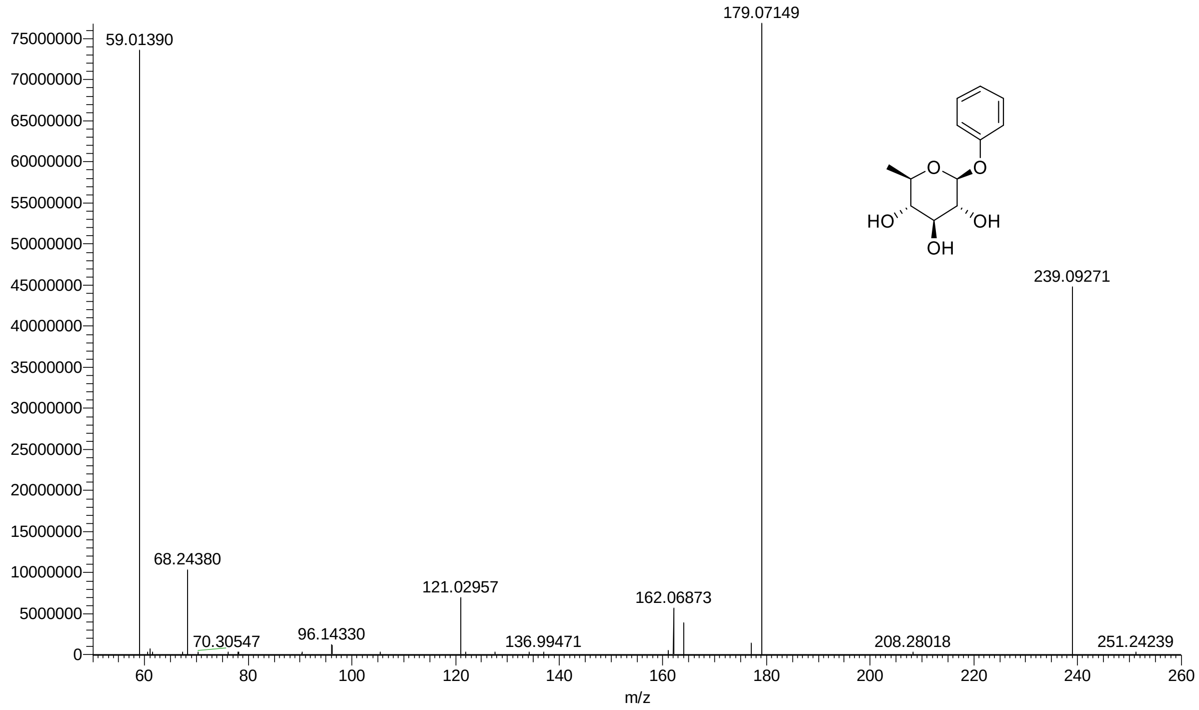

Figure S29. The MS/MS2 spectrum of compound **18.**

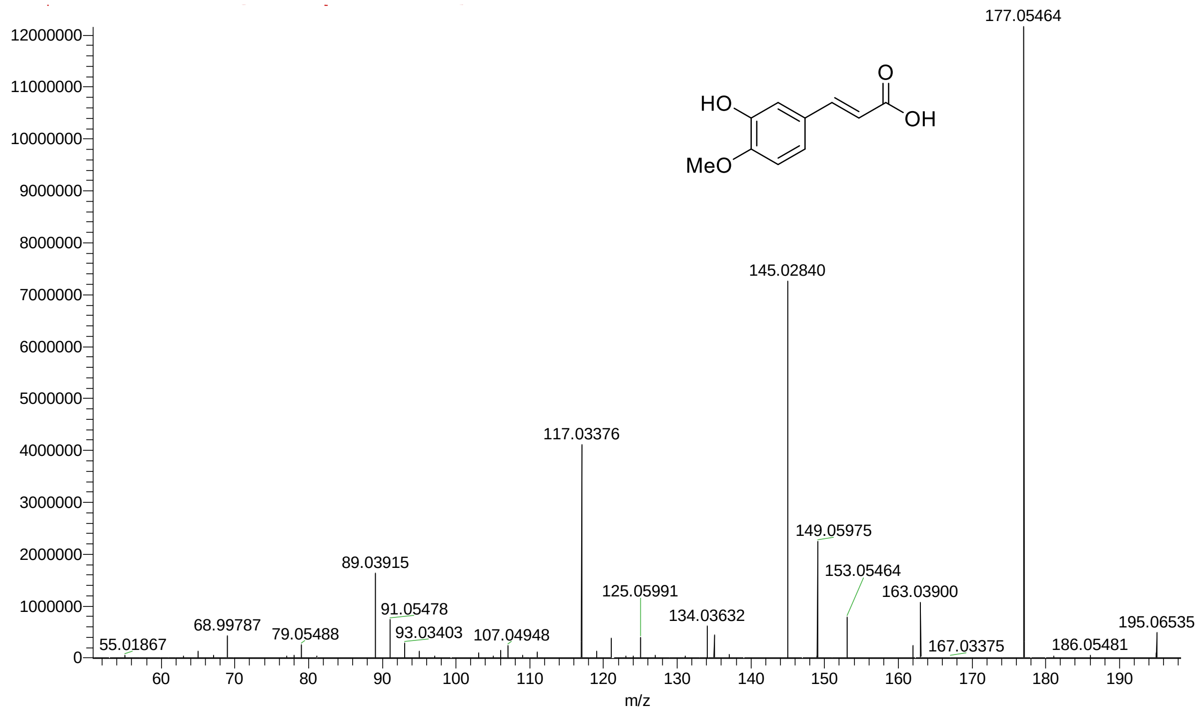

Figure S30. The MS/MS2 spectrum of compound **21.**

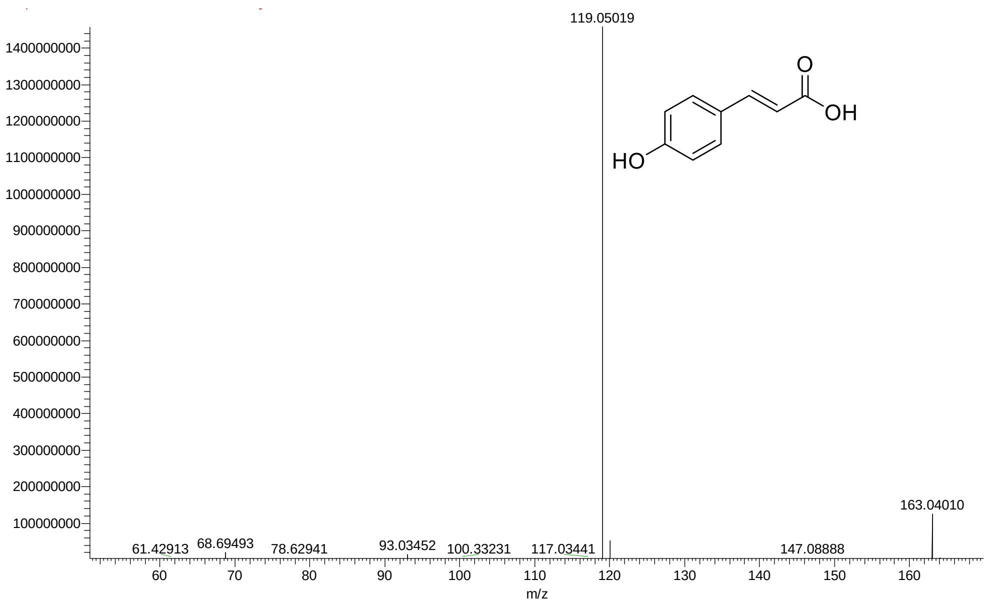

Figure S31. The MS/MS2 spectrum of compound **23.**

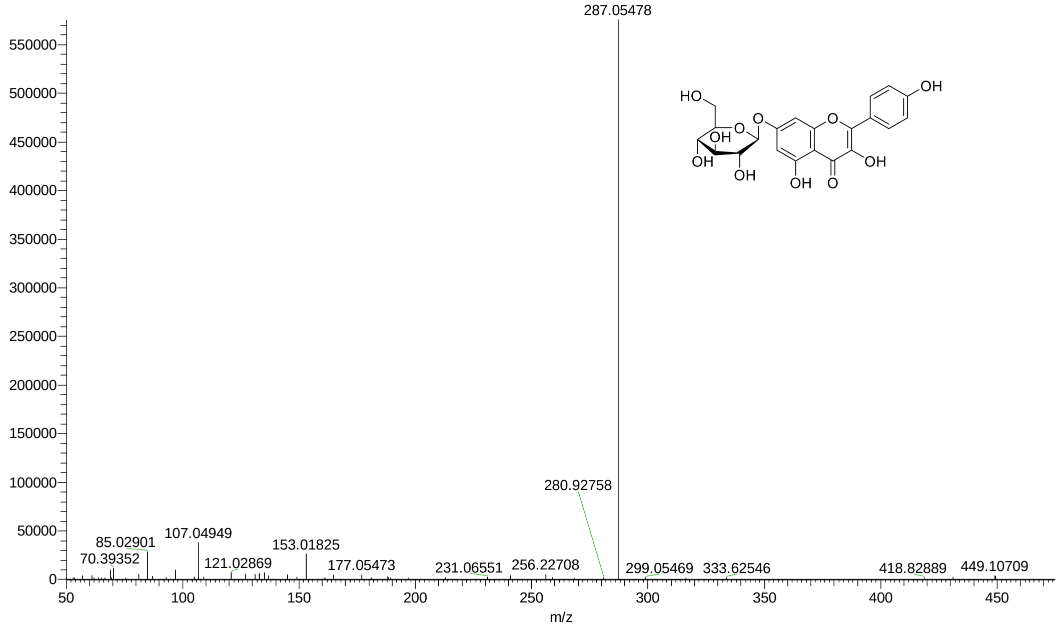

Figure S32. The MS/MS2 spectrum of compound **56.**

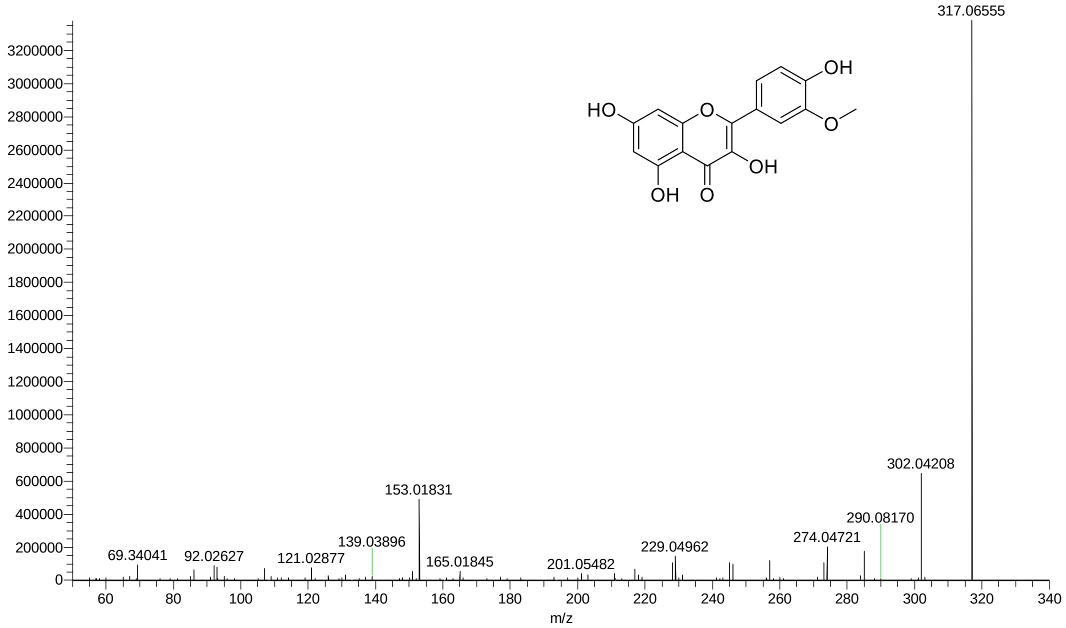

Figure S33. The MS/MS2 spectrum of compound **61.**

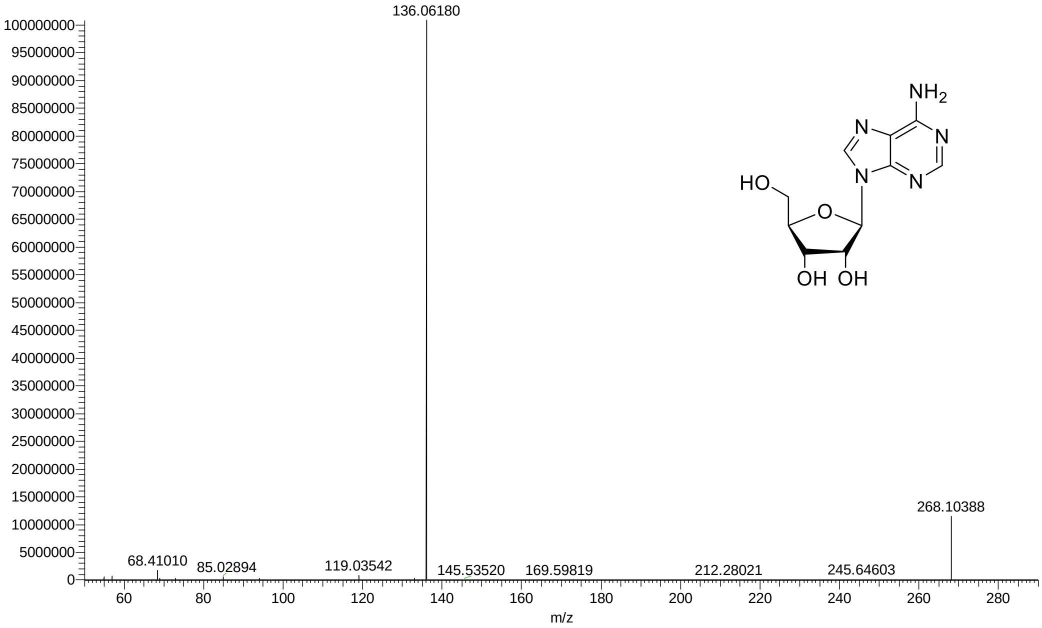

Figure S34. The MS/MS2 spectrum of compound **3.**

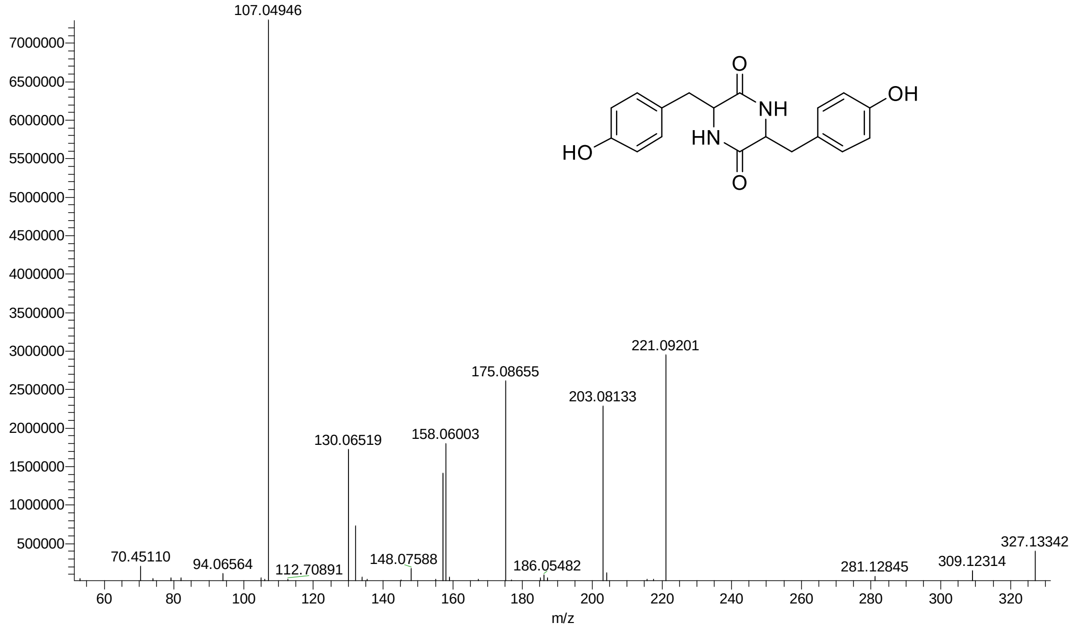

Figure S35. The MS/MS2 spectrum of compound **19.**

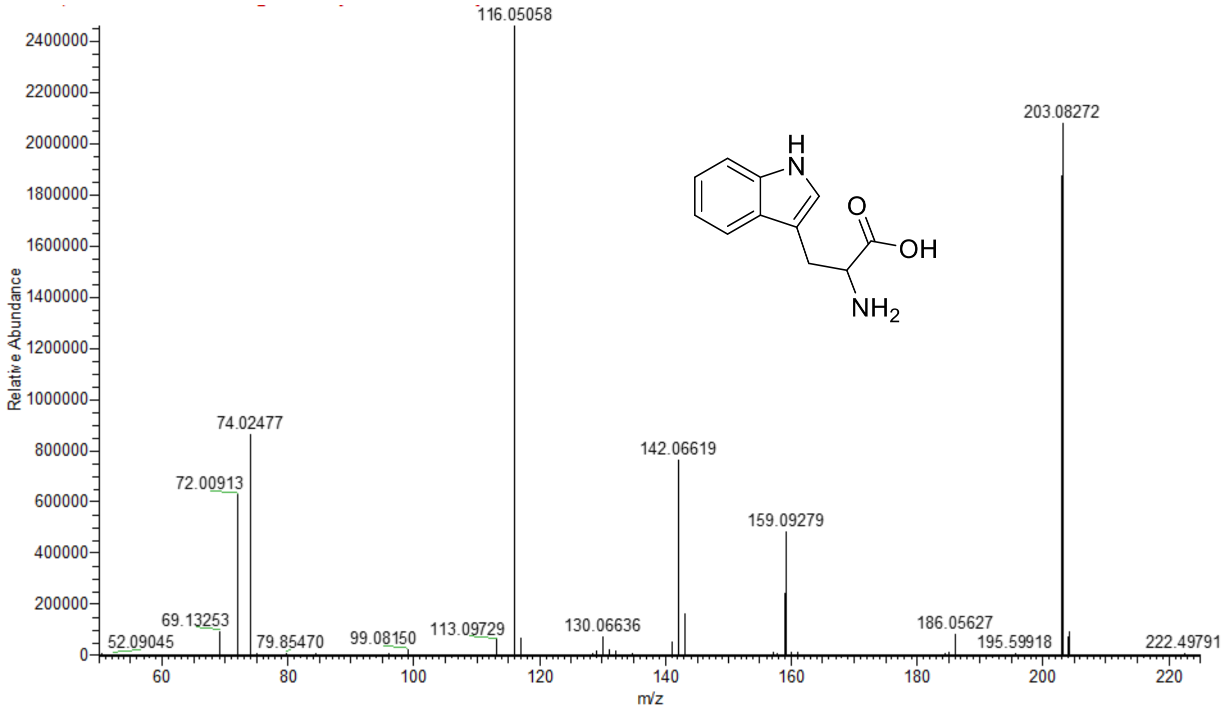

Figure S36. The MS/MS2 spectrum of compound **58.**

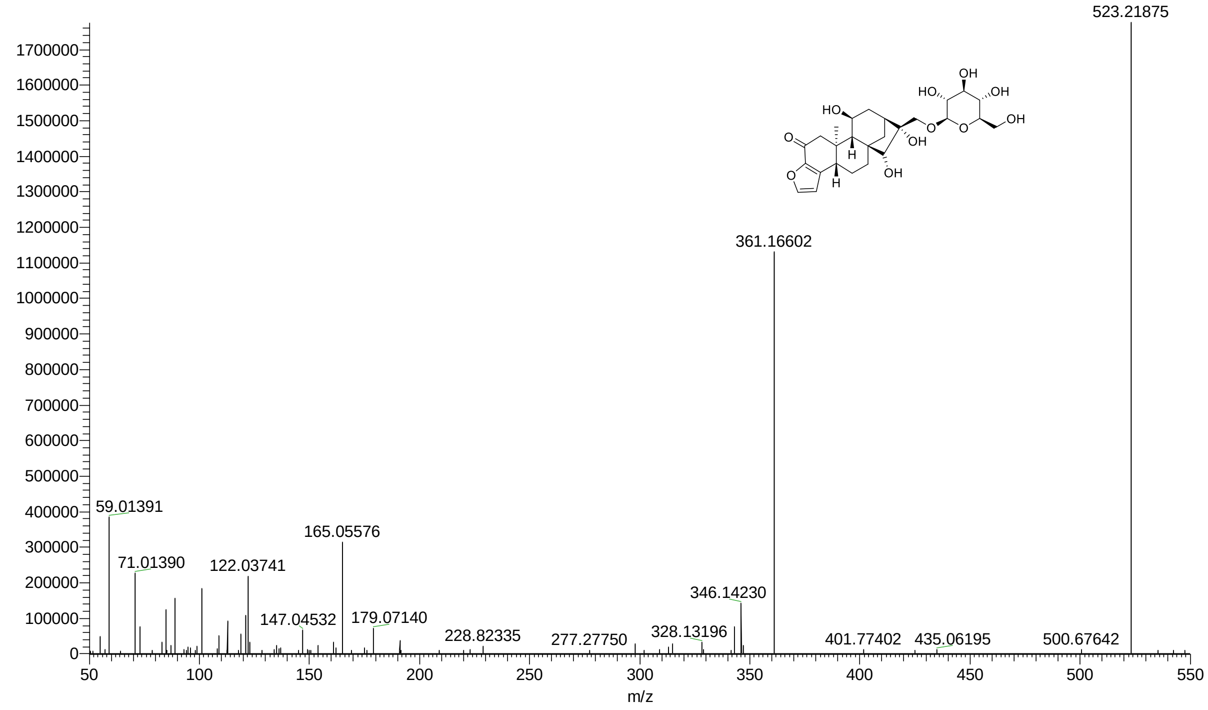

Figure S37. The MS/MS2 spectrum of compound **41.**

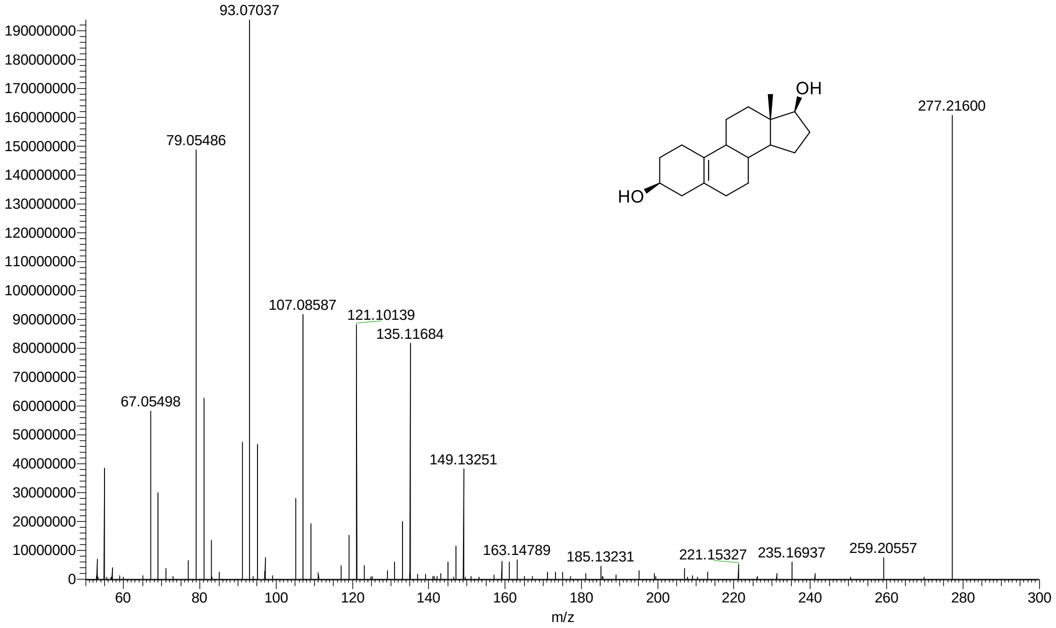

Figure S38. The MS/MS2 spectrum of compound **80.**

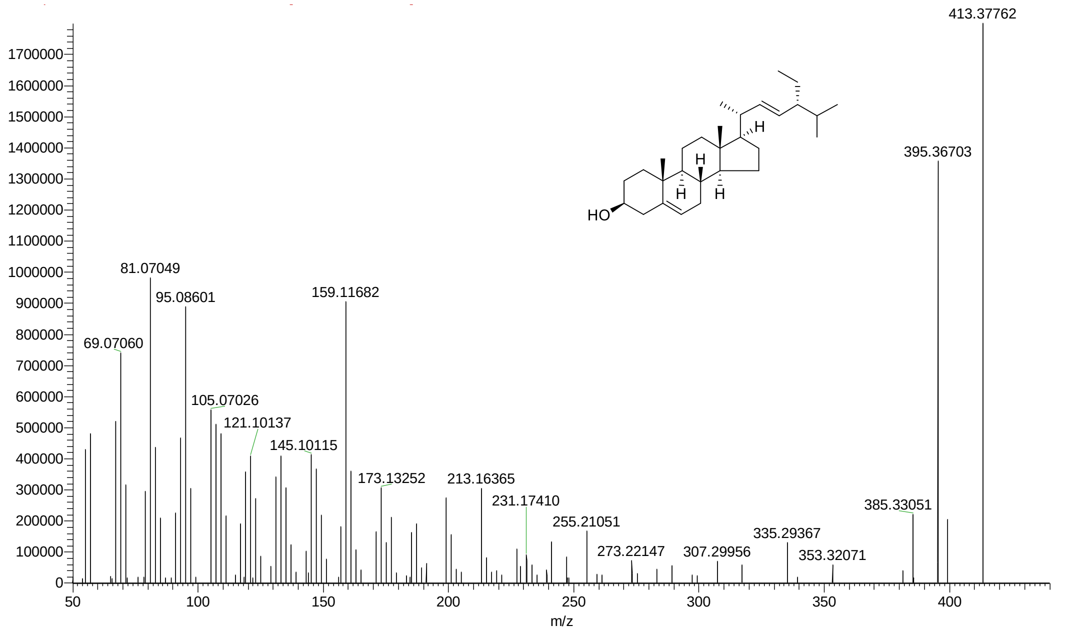

Figure S39. The MS/MS2 spectrum of compound **88.**

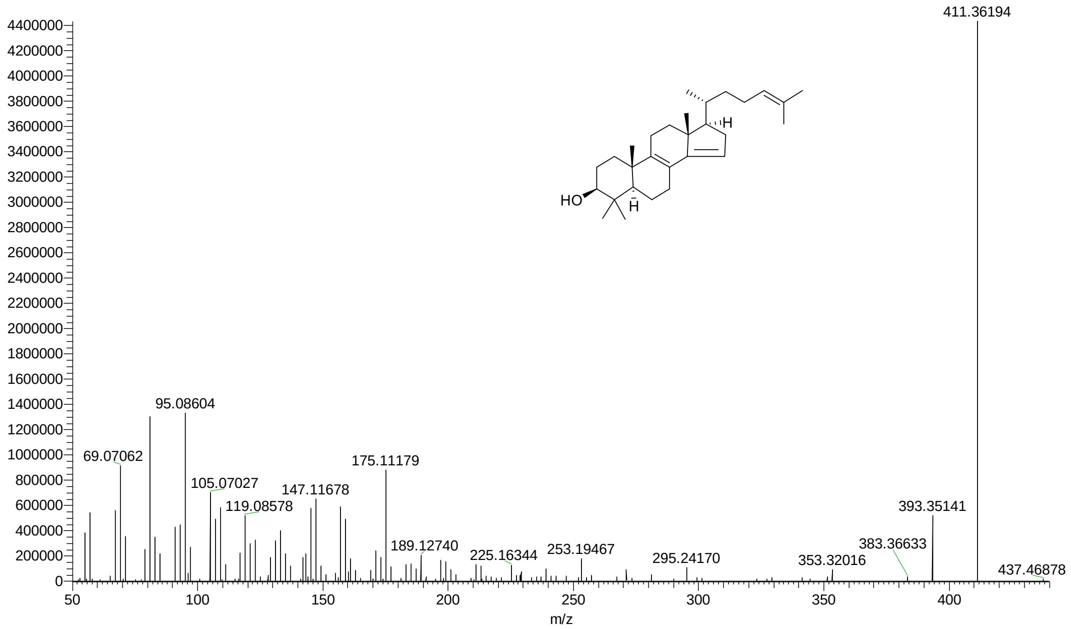

Figure S40. The MS/MS2 spectrum of compound **90.**

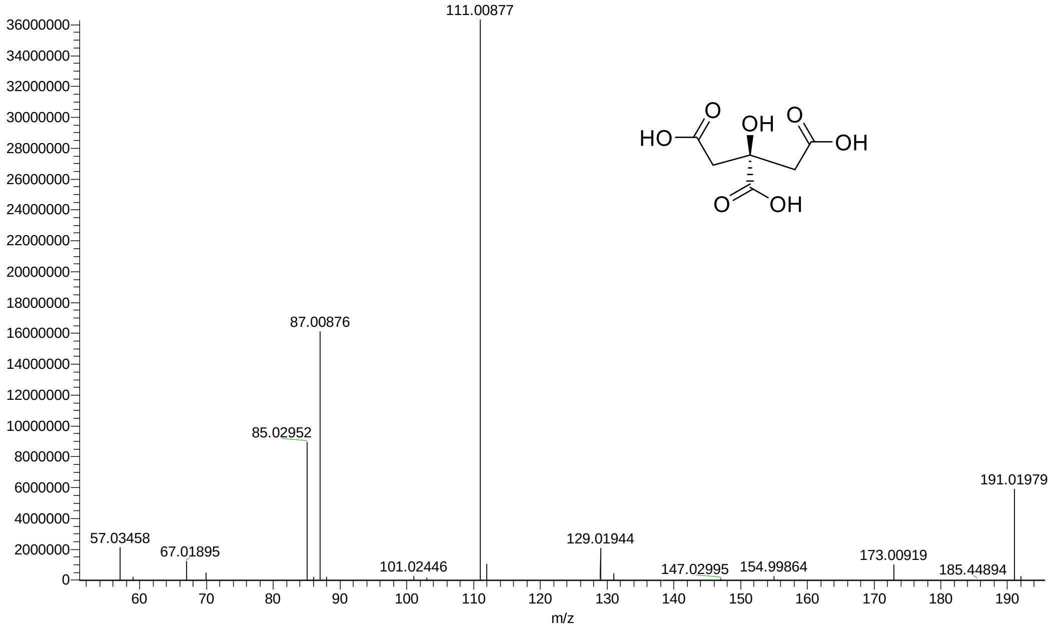

Figure S41. The MS/MS2 spectrum of compound **2.**

The main Compound Discoverer 2.1 Parameter Setting

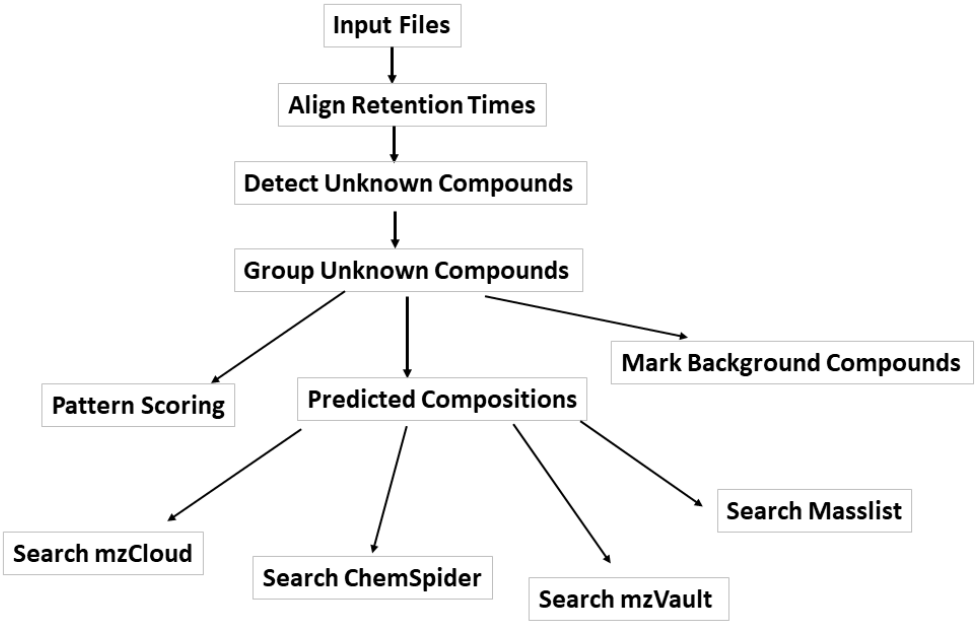

Figure S42. The Workflow tree in Compound Discoverer2.1.

1. Detect Unknown Compounds paraments

The Mass Tolerance was set as 3 ppm; the S/N Threshold was 3; minimum peak intensity was 100000; minimum element counts was C and H; Max. element counts was C100, H190, N10, O50, P, S2, Cl2

2. Group Unknown Compounds paraments

The Mass Tolerance was 3 ppm; RT Tolerance [min] was 0.1; Preferred Ions was [M + H] + 1 and [M − H] − 1.

3. Predict Compositions paraments

Min. RDBE was 0; Max. RDBE was 40; Min. H/C was 0.1; Max. H/C 3.5; Max. Candidates was 10; Pattern Matching Intensity Tolerance was 30; S/N Threshold was 3; Use fragments match was true; Mass Tolerance was 3 ppm.

4. Search mzCloud paraments

Compound Classes was all; Match ion activation type was true; Ion activation energy tolerance was 20; identity search was HighChem High Res; Match factor threshold was 30.

5. Search ChemSpider paraments

Databases were BioCyc, MassBank; NIST, Natural Chemistry, Group Natural products, et al.

6. Search mzVault paraments

Database were mzVault 2017, OTCML

7. Search Masslist paraments

Database was self-bulit *Gymnadenia* compounds database, which including 158 compounds.
